# Perceived occupational physical fitness priorities and a preliminary conceptual framework for information technology students at selected vocational colleges in Shanxi Province, China: An exploratory cross-sectional mixed-methods study

**DOI:** 10.1371/journal.pone.0362309

**Published:** 2026-11-10

**Authors:** Xiaobin Jin, Xiaoying Cui, Yi Zhao, Yu Hou

**Affiliations:** 1 Department of Physical Education, Yuncheng Advanced Normal College, Yuncheng, Shanxi, China; 2 Department of Nursing, Yuncheng Vocational Nursing College, Yuncheng, Shanxi, China; 3 College of Sports Science, Shenyang Normal University, Shenyang, Liaoning, China; Healthcare and Technology Institute (HCT Academy), PHILIPPINES

## Abstract

Students majoring in information technology are frequently exposed to prolonged sitting, continuous screen use, sustained static postures, repetitive fine motor operations, and high cognitive load during their academic training and future work. These conditions may increase the risk of visual fatigue, neck and lower-back discomfort, physical fatigue, and psychological stress. This study adopted an exploratory cross-sectional mixed-methods design, using questionnaire surveys as the primary method and semi-structured interviews as a supplementary method. Participants included information technology students from selected vocational colleges, teachers whose teaching or research activities were related to vocational education or information technology student training, and personnel from information technology-related enterprises in Shanxi Province, China. The final analytical sample comprised 1,924 questionnaires: 1,863 from students, 37 from teachers, and 24 from enterprise personnel. The results showed that 68.71% of students selected “Frequently” or “Occasionally” when asked about physical discomfort following professional practical operations such as prolonged computer use or equipment debugging. The most frequently selected physical-discomfort categories included dry eyes or blurred vision, cervical or lumbar soreness, physical fatigue, and insufficient endurance. Weekly exercise duration was most commonly reported as 1–3 h/week (52.01%), whereas 14.76% of students selected “Almost no exercise.” Qualitative interviews with students and teachers further suggested limited incorporation of occupation-specific training—such as visual relaxation and protection, musculoskeletal relaxation, postural adjustment, and sedentary behavior interruption—into current educational practice. The teacher sample indicated perceived constraints related to limited access to job-demand information, the availability of occupation-specific training methods and teaching resources, and student engagement. The enterprise sample suggested that some enterprise personnel recognized the potential role of occupational physical fitness in job adaptation and work efficiency; however, their actual involvement in curriculum development and the provision of job-related standards remained limited. Based on literature analysis, expert content validity evaluation, questionnaire survey data, and interview data, this study proposed a preliminary conceptual framework for occupational physical fitness in information technology programs, consisting of general physical fitness, occupation-specific physical fitness, and psychological fitness. These findings provide preliminary contextual evidence that occupation-related curriculum content and school–enterprise collaboration may warrant further investigation in vocational-education contexts similar to those examined in this study. Because this study used convenience sampling, included relatively small teacher and enterprise samples, and has not yet verified the proposed framework through structural validity testing or objective measurements, the framework requires further examination in larger and more balanced samples.

## 1. Introduction

With the rapid development of the information technology industry, the learning and working patterns of information technology professionals have shown distinct occupational characteristics, including prolonged sitting, continuous screen viewing, sustained static postures, repetitive keyboard and mouse operations, and high cognitive pressure arising from project-based tasks and employment competition. Although these characteristics differ from those of physically demanding occupations such as policing, firefighting, nursing, and manufacturing, they may still lead to a series of occupational health problems, including visual fatigue, neck and lower-back discomfort, physical fatigue, reduced attention, and increased psychological stress [1–3]. For students majoring in information technology in vocational colleges, these problems may affect not only their current learning efficiency but also their future job adaptation and sustainable employability. Therefore, it is of practical significance to focus on the occupational physical fitness of information technology students at the stage of vocational education.

In this study, occupational physical fitness refers to the integrated manifestation of the physical abilities, occupation-specific work tolerance, and psychological fitness required for an individual to adapt to specific occupational tasks. Unlike general physical fitness, occupational physical fitness emphasizes compatibility with specific job contexts. This is consistent with the perspective in the field of occupational health that “work ability” reflects the match between individual health, physical function, psychological resources, and job demands [4]. From an occupational-health and ergonomic load–capacity perspective, information technology tasks combine prolonged sitting with sustained static postural loading, repetitive fine-motor activity, continuous visual demands, and sustained cognitive demands [1,2,5^–^10]. Accordingly, the present framework distinguishes general physical capacity from task-specific tolerance and psychological regulation: general physical fitness is treated as a broad physiological foundation, whereas sedentary tolerance, visual load tolerance, fine-motor endurance, static postural endurance, and sustained concentration represent capacities more directly aligned with the postural, repetitive, visual, and cognitive demands of information technology tasks. For information technology majors, occupational physical fitness includes not only general physical fitness components such as cardiorespiratory endurance, muscular strength, flexibility, and bodily coordination, but also occupation-specific physical fitness components such as sedentary tolerance, visual load tolerance, fine motor operation endurance, and static postural endurance. It also includes psychological fitness components such as stress resistance, sustained concentration, emotional regulation, and emergency response capacity. Therefore, occupational physical fitness for information technology majors should not be simply understood as general sports ability; rather, it should be regarded as a comprehensive ability structure closely related to professional learning, job adaptation, and career development.

At present, research on occupational physical fitness has mainly focused on occupational groups with relatively obvious physical demands, such as police officers, firefighters, nurses, and manufacturing workers. By contrast, the information technology industry is often regarded as a sector dominated by mental work, and its occupational physical fitness needs are easily overlooked. However, sedentary behavior has been recognized as an important health risk factor independent of moderate-to-vigorous physical activity, and prolonged occupational sitting has been associated with metabolic health, mortality risk, and mental health problems [5–8]. A recent study of software and information technology workers in China also showed that this group had prolonged occupational sitting time on workdays, indicating that information technology jobs are characterized by substantial sedentary exposure [9]. According to the terminology consensus of the Sedentary Behaviour Research Network, sedentary behavior generally refers to any waking behavior characterized by an energy expenditure of no more than 1.5 METs while in a sitting, reclining, or lying posture. Therefore, long-term computer-based learning and office work represent typical screen-based sedentary behavior contexts [11]. Existing studies have paid more attention to single issues such as sedentary behavior, occupational health risks, or psychological stress, whereas relatively few have systematically examined the physical and psychological abilities required by information technology professionals from the perspective of an occupational physical fitness structure. Meanwhile, in the field of vocational education, physical education and health curricula are often centered on general physical exercise and are insufficiently connected with the abilities required by information technology positions, such as sedentary tolerance, visual protection, postural control, and sustained concentration. Previous studies have shown that digital eye strain, or computer vision syndrome, is a common problem among long-term users of digital devices and is manifested by symptoms such as dry eyes, blurred vision, eye fatigue, and headache [1,12–14^]^. In addition, few existing studies have simultaneously incorporated the perspectives of students, teachers, and enterprises, making it difficult to comprehensively reveal the relationships among stakeholder-perceived occupational physical fitness priorities, school-based training provision, and enterprise job requirements.

Although sedentary behavior, digital eye strain, musculoskeletal discomfort, and occupational stress have received considerable attention in occupational health research, most relevant studies have focused on general working populations or current employees, with relatively limited attention paid to information technology students in vocational colleges who are in the transitional stage from learning to internship and employment. Compared with employees, vocational college students’ physical activity habits, postural management patterns, stress regulation strategies, and occupational health awareness are still developing and are therefore highly modifiable. Accordingly, identifying perceived occupational physical fitness priorities and related training conditions among information technology students during vocational education can help clarify their current physical and psychological adaptation problems in learning contexts and provide early evidence for future job adaptation and occupational health education.

There are still three main limitations in the existing literature. First, studies on occupational physical fitness have primarily focused on physically demanding occupations such as policing, firefighting, nursing, and manufacturing, while insufficient attention has been paid to information technology programs characterized by prolonged sitting, continuous screen exposure, and high cognitive load. Second, previous studies have generally discussed sedentary behavior, visual fatigue, musculoskeletal discomfort, or psychological stress separately, and there is a lack of a conceptual framework that integrates general physical abilities, job-context-specific physical tolerance, and psychological adjustment capacity [1,6,10,15]. Third, physical education and health curricula in vocational colleges are usually based on general physical exercise and rarely establish direct links with abilities required for information technology positions, such as visual protection, postural management, sedentary behavior interruption, and sustained concentration. Moreover, comprehensive evidence from the perspectives of students, teachers, and enterprises remains limited. Based on these gaps, this study aimed to: (1) describe the current status of occupational physical fitness-related physical discomfort, psychological stress, and training participation among information technology students at selected vocational colleges in Shanxi Province, China; (2) compare students’, teachers’, and enterprise personnel’s importance ratings of occupational physical fitness dimensions and core indicators, interpreted as perceived occupational physical fitness priorities in information technology programs; and (3) propose a preliminary conceptual framework of occupational physical fitness applicable to vocational-education contexts examined in this study, based on literature, expert evaluation, questionnaire surveys, and interview data.

## 2. Methods

### 2.1. Study design

This study adopted a quantitative-dominant cross-sectional mixed-methods design, with a quantitative questionnaire survey as the primary research method and semi-structured interviews as a supplementary explanatory qualitative component. The quantitative survey was used to describe self-reported physical discomfort, self-reported psychological experiences, training participation, training- and provision-related conditions, and evaluations of the core indicators of occupational physical fitness among students, teachers, and enterprise personnel. The qualitative interviews were used to provide supplementary contextual explanation of questionnaire findings. The quantitative and qualitative strands were analyzed separately and subsequently integrated at the interpretation stage. Integration focused on whether the documented qualitative themes complemented, contextualized, or qualified quantitative findings concerning student-reported experiences, stakeholder-rated priorities, and provision constraints. The qualitative findings were not converted into quantitative variables or used for statistical inference.

The mixed-methods design and integration were described with reference to established mixed-methods methodological principles [16]. Reporting of the quantitative cross-sectional component was prepared with reference to the STROBE statement [17,18]. Reporting of the semi-structured interview component was retrospectively reviewed against the Consolidated Criteria for Reporting Qualitative Research (COREQ) to improve reporting transparency [19]. Because the original interview materials and detailed procedural records were no longer available at the time of revision, COREQ items that could not be verified from the contemporaneous study-report documentation were identified as unavailable or not documented rather than retrospectively reconstructed. A completed COREQ checklist is provided in the Supporting Information (S1 Checklist). A completed STROBE checklist for the quantitative cross-sectional component is provided in the Supporting Information (S2 Checklist).

### 2.2. Participants and sample sources

The participants were students majoring in information technology at selected vocational colleges, teachers whose teaching or research activities were related to vocational education or the training of information technology students, and personnel from information technology-related enterprises in Shanxi Province, China. The teacher sample included participants from vocational and other higher-education institutions; eligibility was based on the relevance of their teaching or research activities to vocational education or information technology talent development rather than on institution type alone.

Participants were recruited using a combination of convenience sampling and snowball sampling. The student sample was mainly recruited through class WeChat groups, course groups, and student communities in vocational colleges. The teacher sample was recruited through teacher work groups and institutional contacts. The enterprise sample was recruited through school–enterprise partner companies, industry WeChat groups, and enterprise contacts.

Detailed distributions of students by year/stage of study and information technology field, teachers by institution type and professional background, and enterprise personnel by industry sector and job function are presented in the Results section. However, the analytical dataset did not retain identifiers for individual colleges, departments, classes, companies, cities, or participant-level recruitment channels. Consequently, the exact distribution of participants across these recruitment units could not be retrospectively reconstructed.

The inclusion criteria for students were as follows: (1) being enrolled in an information technology-related major; (2) having certain professional learning or practical experience; (3) voluntarily participating in the study and providing informed consent; and (4) completing the questionnaire in full. The inclusion criteria for teachers were as follows: (1) being engaged in information technology teaching, physical education teaching, or student training and management; (2) being familiar with the training of information technology students; and (3) voluntarily participating in the study. The inclusion criteria for enterprise personnel were as follows: (1) working in software development, cybersecurity, artificial intelligence, cloud computing, or related information technology enterprises; (2) having experience in technical positions, management positions, or talent recruitment; and (3) being familiar with the work characteristics and talent needs of information technology positions.

The exclusion criteria were as follows: (1) a questionnaire completion time of less than 90s; (2) one or more unanswered questionnaire items; (3) extensive long-string responding, characterized by repeated selection of the same response option across a substantial sequence of questionnaire items; (4) potential duplicate submissions identified by repeated IP addresses; and (5) a reported academic major or occupational role with no identifiable connection to information technology-related learning or work. Each excluded questionnaire was assigned only one primary exclusion reason.

A total of 2,106 questionnaires were returned during the survey period. After application of the prespecified data-quality and eligibility criteria, 182 questionnaires were excluded, leaving 1,924 questionnaires in the final analytical sample. A total of 1,924 questionnaires were therefore retained for analysis, including 1,863 from students, accounting for 96.83%; 37 from teachers, accounting for 1.92%; and 24 from enterprise personnel, accounting for 1.25%.

The historical pre-cleaning records required to reconstruct the stakeholder-specific numbers of returned questionnaires and the numbers excluded under each individual screening criterion were no longer available at the time of revision. Similarly, platform-level counts of individuals who accessed the survey, provided consent, or began but did not complete the questionnaire could not be reliably reconstructed. As students are the primary target group of vocational education and training, the student sample size in this study was relatively large. The teacher and enterprise personnel samples were mainly used to provide supplementary explanations regarding school-based provision and job demands. Given the relatively small sample sizes of teachers and enterprise personnel, the corresponding results were interpreted primarily as descriptive and exploratory evidence rather than as a basis for confirmatory population-level inference.

Data were collected from 20/04/2025 to 29/05/2025. Because the participants included three groups—students, teachers, and enterprise personnel—and because it was difficult to randomly select teachers and enterprise personnel using a unified sampling frame, this study adopted a combination of convenience sampling and snowball sampling for recruitment. The student sample was used to describe the current status of occupational physical fitness and related training conditions among information technology students and served as the main analytical sample of this study. The teacher and enterprise samples were used primarily to provide supplementary explanations regarding school-based curriculum provision and job demands. Therefore, the results from the teacher and enterprise samples were not used to make inferences about the overall industry population; instead, they were treated as auxiliary evidence for comparing the perspectives of the three stakeholder groups and for constructing the framework. Given the exploratory nature of this study, the sample size was not estimated a priori based on causal effect testing. Future studies should conduct confirmatory analyses using larger and more balanced samples.

### 2.3. Survey procedure

The online survey was conducted through the Wenjuanxing platform. Before the formal survey, the research team conducted multiple rounds of checks on the questionnaire content, item logic, and completion process. A small-scale pilot test was also conducted to refine the wording of items and the questionnaire structure. During the formal survey period, the questionnaire link was distributed through college class groups, teacher work groups, industry groups, and school–enterprise cooperation contacts. At participating educational institutions, teachers assisted only with forwarding the study invitation and questionnaire link to eligible student groups. The decision to participate was made independently by each student; distributing teachers did not determine, select, or require which individual students participated. Similarly, for enterprise recruitment, designated enterprise contacts assisted only in forwarding the study invitation to potentially eligible personnel and had no role in determining whether individual employees participated.

All participants read the informed consent statement before completing the questionnaire and entered the formal questionnaire only after confirming their voluntary participation. The questionnaire was administered anonymously and did not collect personally identifiable information such as names, identity card numbers, or telephone numbers. The anonymous survey procedure prevented questionnaire responses from being linked to an individual participant for academic or employment-related purposes. The interview-related methodological information reported in this manuscript is based on the contemporaneous study-report documentation available to the research team. The original interview audio files, verbatim transcripts, field notes, and detailed interview-procedure records were no longer available at the time of manuscript revision; therefore, procedural details that were not documented in the surviving study report were not retrospectively inferred.

To provide supplementary explanations for the questionnaire results, this study also conducted semi-structured interviews. Interview participants were purposively selected within the three stakeholder strata—information technology students, teachers, and enterprise personnel—to capture variation in relevant educational and occupational backgrounds and experiences. The qualitative component included 30 interview participants: 21 students, 6 teachers, and 3 enterprise personnel. The qualitative sample was intended to provide contextual variation across stakeholder perspectives rather than statistical representativeness. Detailed individual demographic profiles were not systematically recorded in order to minimize the collection of potentially identifying information. The interviews mainly focused on the current status of occupational physical fitness, experiences of physical discomfort, training participation, problems in curriculum provision, job demands, and suggestions for school–enterprise cooperation. According to the contemporaneous study-report documentation, with participants’ consent, interviews were audio-recorded or documented in written form, and the resulting materials were organized as textual data for thematic analysis.

The interview-related methodological information reported in this manuscript was based on the contemporaneous study-report documentation available to the research team. At the time of manuscript revision, the original interview audio files, verbatim transcripts, field notes, and detailed interview-procedure records were no longer available for independent retrospective verification. Therefore, qualitative reporting details that were not documented in the study report, including interviewer characteristics, exact interview duration, repeat interviews, formal saturation assessment, transcript return, and member checking, could not be reliably reconstructed and were not retrospectively inferred.

### 2.4. Ethics statement

This study involved human participants recruited from postsecondary educational and occupational settings. Student participants were enrolled in vocational-college information technology programs. Under the standard Chinese educational progression, children generally enter primary education at six years of age and complete 12 years of basic education before entering higher education. Data collection was conducted from April 20 to May 29, 2025; therefore, even first-year students who had matriculated in September 2024 had progressed beyond the first semester of postsecondary education. On this basis, student participants were expected to be at least 18 years of age at the time of data collection. However, because the anonymous student questionnaire did not collect age or date of birth, individual participant age was not independently verified and cannot be retrospectively confirmed from the analytical dataset. It was reviewed and approved by the Ethics Committee of Yuncheng Advanced Normal College, with the approval number 2025−0013 and the approval date of April 18, 2025. All participants read the study information and informed consent materials before completing the questionnaire or participating in the interviews. Participants in the online questionnaire entered the formal questionnaire after clicking “I agree to participate in this study,” and interview participants confirmed their voluntary participation before the interviews. Participation was entirely voluntary. For student participants, participation or non-participation had no effect on course grades, academic standing, access to educational opportunities, or other academic treatment, and teachers who distributed the invitation had no authority to require participation. Students could decline the invitation without providing a reason and without any adverse consequences. For enterprise personnel, participation or non-participation similarly had no effect on employment-related standing or workplace treatment, and enterprise contacts who forwarded the invitation had no authority to require employees to participate. Employees were likewise free to decline without providing a reason or experiencing any adverse consequences.

Data were collected anonymously, and no directly identifiable personal information, such as names, identity card numbers, or telephone numbers, was collected. All data were used only for academic research and were anonymized during data processing and analysis. Participants were free to withdraw from the study at any time before submitting the questionnaire or during the interviews, without any adverse consequences.

### 2.5. Inclusivity in global research

Additional information regarding the ethical, cultural, and scientific considerations specific to inclusivity in global research is included in the Supporting Information (S3 Checklist).

### 2.6. Questionnaire design

This study used a self-developed questionnaire entitled Questionnaire on Occupational Physical Fitness Qualities and Training Needs of Information Technology Students. The questionnaire was developed in four steps. First, the research team established an initial item pool based on literature related to occupational physical fitness, occupational health, sedentary behavior, characteristics of information technology positions, and vocational education curriculum reform. Second, in accordance with the learning and working contexts of information technology programs, three dimensions were determined: general physical fitness, occupation-specific physical fitness, and psychological fitness. Third, eight experts in the fields of physical education, vocational education, information technology teaching, and enterprise management were invited to evaluate the content validity of the questionnaire items and indicator framework. The experts evaluated the items mainly in terms of item relevance, clarity of wording, professional applicability, and rationality of dimensional assignment, and provided suggestions on whether each indicator was suitable for inclusion in the occupational physical fitness framework for information technology programs. Expert evaluation was conducted using a four-point rating scale, where 1 indicated not relevant, unclear, or not applicable, and 4 indicated highly relevant, very clear, or highly applicable [20]. Based on expert feedback, the research team revised the wording of some items, indicator names, and dimensional assignments. Fourth, a small-scale pilot test was conducted to further examine the questionnaire logic, language expression, and comprehensibility, after which the final version of the questionnaire was developed.

The questionnaire included three versions for students, teachers, and enterprise personnel. Stakeholder-specific sections were adapted to the educational or occupational context of each group; however, the 12 core occupational physical fitness indicators used for cross-stakeholder comparison were administered with identical indicator names, descriptions, five-point importance-response options, and scoring direction across all three versions. The introductory stem preceding the 12 core items was contextually adapted so that students responded from the perspective of their learning experience and future work, teachers from the perspective of students’ future career development, and enterprise personnel from the perspective of actual job demands and employee work performance. A side-by-side mapping of the common core items, response scales, and stakeholder-specific introductory stems across the three questionnaire versions is provided in S1 Table.

No cross-group statistical comparisons were conducted for stakeholder-specific questionnaire items that differed in wording or content across the three versions. A side-by-side mapping of the three questionnaire versions and the common core items is provided in the Supporting Information.

The questionnaire mainly consisted of five sections: basic information, perceptions of occupational physical fitness, current status of occupational physical fitness, training needs, and open-ended suggestions. The core content was designed around the three-dimensional structure of “general physical fitness–occupation-specific physical fitness–psychological fitness” and included 12 core indicators of occupational physical fitness. The specific indicator composition, scoring method, and calculation method for dimensional scores are described in Section 2.6. In addition to closed-ended items, the questionnaire included open-ended questions to collect participants’ supplementary opinions on occupational physical fitness training.

Background characteristics were collected separately according to stakeholder group. The student questionnaire collected gender, year/stage of study, information technology field, weekly professional practical-operation time, information technology-related internship experience, and weekly exercise duration. Student age and specific institution identity were not collected in the questionnaire. The teacher questionnaire collected gender, age group, institution type, teaching experience, professional title, and main teaching field. The enterprise questionnaire collected industry sector, enterprise size, the number of vocational-college information technology graduates recruited during the previous three years, and the respondent’s main area of responsibility. Individual age, gender, and years of professional experience were not collected from enterprise respondents. In the student questionnaire, the pre-graduation category referred to students who had completed their required coursework and were awaiting formal issuance of their graduation certificates; they had not formally graduated at the time of participation.

A complete data dictionary for the final analytical dataset, including exact variable names and labels, response coding, identification of multiple-response variables, applicable stakeholder groups and denominators, structural missing-data conventions, and data-processing notes, is provided in S2 Table.

It should be noted that the questionnaire used in this study was developed by the research team according to the study purpose and the context of information technology programs. It was mainly used to characterize stakeholder-perceived occupational physical fitness priorities and related physical, psychological, training, and provision conditions and to construct a preliminary conceptual framework. Therefore, the results of reliability and content validity should be interpreted as preliminary measurement evidence rather than as complete psychometric validation of a mature scale.

### 2.7. Variables, measurement, and occupational physical fitness indicator framework

The main variables in this study included perceptions of occupational physical fitness, self-reported physical discomfort, participation in physical exercise, participation in occupation-specific training, self-reported psychological experiences, school-based curriculum provision, enterprise involvement, and importance ratings of core indicators of occupational physical fitness. Physical discomfort mainly referred to whether students experienced symptoms such as dry eyes or blurred vision, cervical or lumbar soreness, physical fatigue, and insufficient endurance after learning or practice in information technology programs. Participation in physical exercise mainly included weekly exercise duration and common forms of exercise. Participation in occupation-specific training mainly included training content related to information technology learning and future job contexts, such as visual relaxation and protection training, musculoskeletal relaxation training for the neck, shoulder, and lower-back regions, postural adjustment training, and sedentary behavior interruption training. Self-reported psychological experiences were assessed using questionnaire items concerning experiences during professional learning or practice, including nervousness related to employment or internship performance, sleep being affected before examinations or project deadlines, difficulty maintaining concentration after prolonged study or programming, irritability when encountering technical problems, and other listed experiences. These questionnaire items were developed for the present exploratory study and were not derived from validated clinical instruments for anxiety, sleep disturbance, or other mental health conditions. Accordingly, they were interpreted as self-reported experiences rather than as clinical symptoms, diagnoses, or measures of psychological disorders.

Similarly, the physical-discomfort items were self-developed descriptive questions rather than validated diagnostic instruments for digital eye strain or musculoskeletal disorders; therefore, the corresponding findings were interpreted according to the specific symptoms reported by participants.

The principal student-reported outcomes were analyzed according to the wording and response categories used in the original questionnaire. For physical discomfort (QB8), students were asked, “After professional practical operations, such as long-term computer use or equipment debugging, do you experience physical discomfort?” The response categories were “Frequently,” “Occasionally,” “Rarely,” and “Never.” The questionnaire did not specify a fixed retrospective recall period or numerical frequency thresholds for these four categories. For descriptive reporting, the categories “Frequently” and “Occasionally” were combined where explicitly indicated; this grouping represents a descriptive combination of questionnaire responses rather than a validated frequency threshold.

Physical discomfort symptoms (QB9) were assessed using a multiple-response item among the listed symptom categories, including cervical or lumbar soreness, dry eyes or blurred vision, soreness or swelling of the wrist or finger joints, dizziness or difficulty concentrating, and physical fatigue or insufficient endurance, with “None” also provided as a response option. No additional retrospective recall period was specified beyond the context of discomfort following professional practical operations. “Cervical or lumbar soreness” was presented as a single combined response option in QB9; therefore, cervical and lumbar symptoms could not be separated analytically, and no site-specific response proportion was calculated for either region individually. Throughout the manuscript, this questionnaire-derived category is reported using the exact wording of the original item.

Psychological experiences (QB10) were assessed using a multiple-response question asking whether students had experienced specified psychological states during professional learning or practice. The item included context-specific experiences such as anxiety affecting sleep before examinations or project deadlines, inability to concentrate after approximately two hours of continuous study or programming, irritability when encountering technical problems, and nervousness concerning employment or internship performance. No uniform retrospective recall period was specified for this multiple-response item. Accordingly, the reported percentages represent the proportions of respondents selecting each response option and are not period-prevalence estimates.

Weekly exercise duration (QB6), in contrast, was explicitly assessed as average exercise time per week, with response categories of almost no exercise, 1–3 h/week, 3–5 h/week, 5–7 h/week, and >7 h/week. Because QB6 assessed exercise duration only and did not separately measure exercise intensity, frequency, moderate-to-vigorous physical activity, or sedentary time, these response categories were not used to determine whether participants met established physical-activity recommendations. No threshold was therefore applied to classify students as having “sufficient” or “insufficient” physical activity.

The importance ratings of the core indicators of occupational physical fitness were measured using a five-point Likert scale, where 1 indicated “not important at all” and 5 indicated “very important.” The 12 core indicators were organized around three dimensions: general physical fitness, occupation-specific physical fitness, and psychological fitness. General physical fitness refers to the general physical abilities that support students’ long-term learning and future occupational adaptation, including cardiorespiratory endurance, muscular strength, flexibility, and bodily coordination. Occupation-specific physical fitness refers to physical tolerance capacities directly related to the learning and working contexts of information technology programs, including sedentary tolerance, visual load tolerance, fine motor operation endurance, and static postural endurance. Psychological fitness refers to the psychological capacity to maintain stable performance in the contexts of professional learning, project-based tasks, employment competition, and job-related stress, including stress resistance, sustained concentration, emotional regulation, and emergency response capacity.

For the cross-stakeholder analyses, each dimension score was calculated from the same four core indicators in each stakeholder group. Because the item labels, item descriptions, response categories, coding direction, and score calculation were identical across the three versions, the resulting scores represented the same nominal construct of perceived importance. However, because the introductory stems were stakeholder-specific and formal measurement invariance was not evaluated, these scores should not be interpreted as demonstrating full psychometric equivalence across groups. Cross-group comparisons were therefore considered exploratory.

The score for each dimension was calculated as the mean score of the indicators included in that dimension, with higher scores indicating that respondents perceived the corresponding dimension or indicator as more important.

In this study, “perceived occupational physical fitness priorities” refers specifically to the relative importance assigned by participants to the occupational physical fitness dimensions and core indicators using the five-point importance-rating scale. Higher scores therefore indicate greater perceived importance or priority, rather than a directly measured unmet need. Self-reported physical discomfort and psychological experiences were treated as experienced conditions; exercise and occupation-specific training participation were treated as behavioral or training-status variables; and school-based curriculum provision and enterprise involvement were treated as contextual provision variables. These constructs were analyzed separately and were not assumed to represent interchangeable measures of a single latent need.

The indicator framework was developed primarily based on the occupational characteristics of information technology programs, including prolonged sitting, continuous screen exposure, sustained static postures, repetitive fine motor operations, and relatively high cognitive load. General physical fitness provides the general physical foundation for occupational adaptation; occupation-specific physical fitness reflects the contextualized demands of information technology positions; and psychological fitness reflects students’ capacity for sustained performance under academic tasks, project pressure, and occupational competition. It should be noted that the 12 core indicators in this study were mainly used to characterize perceived occupational physical fitness priorities and to support the construction of a preliminary conceptual framework in information technology programs and to support the construction of a preliminary conceptual framework, rather than representing a fully validated mature scale structure. Therefore, the related scoring results should be interpreted as exploratory measurement evidence, and the structural validity of the framework requires further examination in future research. To improve measurement transparency, the exact questionnaire wording, temporal or contextual reference frames, response categories, and scoring or reporting rules for the principal outcomes are summarized in Supplementary S3 Table. Where the original questionnaire did not specify a fixed recall period or numerical frequency threshold, no such definition was assigned retrospectively.

### 2.8. Internal consistency testing and expert content validity evaluation

This study examined the internal consistency of the importance ratings for the 12 core indicators of occupational physical fitness. Cronbach’s α was used to assess the degree of consistency among items within the same preliminary conceptual framework and is a commonly used indicator of internal consistency in questionnaire and scale research [21,22]. Because the 12 indicators in this study were mainly used to construct a preliminary conceptual framework of occupational physical fitness rather than as reflective items of a mature scale, the Cronbach’s α results were used only as exploratory evidence of internal consistency within the preliminary conceptual framework and not as reliability evidence for a mature scale. Factor-analytic or latent-variable modeling was not part of the original analysis plan because the importance-rating items were used as an a priori conceptual indicator set rather than as a finalized psychometric scale with an established reflective measurement model.

Content validity was evaluated using expert judgment. The research team invited eight experts in physical education, vocational education, information technology teaching, and enterprise management to rate the relevance, clarity, applicability, and rationality of dimensional assignment of the 12 core indicators of occupational physical fitness. Expert ratings were conducted using a four-point rating scale, where 1 indicated not relevant or not applicable and 4 indicated highly relevant or highly applicable. Based on the expert ratings, the item-level content validity index (I-CVI) was calculated, and the scale-level average content validity index for the overall preliminary conceptual framework (S-CVI/Ave) was further computed. The I-CVI was obtained by calculating the proportion of experts who rated each indicator as 3 or 4 among the total number of experts, while the S-CVI/Ave was obtained by calculating the mean of the I-CVI values across all indicators [20].

### 2.9. Analysis of interview data

According to the contemporaneous study-report documentation, the interview data were analyzed using thematic analysis, a method suitable for identifying, analyzing, and presenting recurring patterns of meaning within interview texts [23]. The research team first organized the interview recordings or written records into textual data. Subsequently, two researchers independently read the interview texts and extracted initial codes related to perceptions of occupational physical fitness, experiences of physical discomfort, training barriers, difficulties in curriculum provision, enterprise job demands, and suggestions for school–enterprise cooperation. The researchers then compared and discussed the initial codes and merged similar codes into themes. Discrepancies in coding were resolved through discussion until consensus was reached. Three main themes were ultimately identified: occupational physical fitness problems among students and the lack of training methods; insufficient institutional resources for occupational physical fitness training; and inadequate alignment between enterprise job demands and school-based training provision. The interview results were mainly used to supplement and explain the phenomena identified in the questionnaire survey.

At the time of manuscript revision, the original interview audio files, verbatim transcripts, field notes, detailed procedural records, and original coding records were no longer available for independent retrospective verification. Accordingly, the qualitative findings reported here are based on the thematic results documented in the contemporaneous study report. Interview-procedure or analytic details that were not documented in the surviving study report could not be reliably reconstructed and were therefore not retrospectively inferred.

The qualitative findings were treated as supplementary explanatory and contextual evidence rather than as independently revalidated qualitative data or as a basis for statistical inference. After the quantitative and qualitative findings had been considered separately, they were integrated at the interpretation stage by examining how the documented interview themes complemented, contextualized, or qualified the questionnaire findings. The integrated interpretation was organized around three conceptually distinct domains: student-reported experiences, stakeholder-rated priorities, and provision constraints. A detailed joint display linking the quantitative findings, qualitative themes, and integrated interpretations is provided in S4 Table. Interview participant characteristics and the three documented themes are summarized in Table 1.

**Table 1:** Summary of interview participants and themes.

Participant Group	Number of Interviewees	Corresponding theme	Theme
Student group	21	Occupational physical fitness problems among students and lack of training methods	“I do not know how to exercise specifically to relieve eye fatigue.”
Teacher group	6	Insufficient institutional resources for occupational physical fitness training	“There is a lack of job demand data, so the curriculum cannot be designed precisely.”
Enterprise personnel group	3	Inadequate alignment between enterprise job demands and school-based training provision	“Technical positions require long periods of concentration, so endurance and stress resistance are critical.”

Interview participants were purposively selected across the student, teacher, and enterprise stakeholder groups to capture variation in relevant educational and occupational experiences. Detailed individual demographic characteristics were not systematically recorded. Representative quotations shown here were preserved in the contemporaneous study-report documentation; because the original audio recordings and verbatim transcripts were no longer available at the time of revision, they could not be independently rechecked against the original interview materials.

### 2.10. Statistical analysis

Data analysis was performed using SPSS 26.0 and Excel 2016. Before analysis, the research team screened the questionnaire data using predefined data-quality and eligibility criteria. Questionnaires with a completion time of less than 90 s, one or more unanswered items, extensive long-string responding characterized by repeated selection of the same response option across a substantial sequence of items, repeated IP addresses indicating potential duplicate submissions, or a reported academic major or occupational role unrelated to information technology were excluded. Each excluded questionnaire was assigned only one primary exclusion reason. No imputation was performed because questionnaires containing unanswered items were excluded during data screening. Of the 2,106 returned questionnaires, 182 were excluded during screening and 1,924 were retained for analysis. Because this study mainly used descriptive statistics and non-parametric between-group comparisons, extreme values were not manually replaced. If abnormal values beyond the theoretical range of a variable were identified, the original questionnaire records were checked, and such values were treated as invalid data when appropriate.

First, descriptive statistics were used to summarize the basic characteristics of the sample, physical discomfort, participation in exercise, perceptions of occupational physical fitness, school-based training provision, and enterprise involvement. Categorical variables were expressed as frequencies and percentages. Continuous variables or Likert-scale variables were described using means, standard deviations, medians, and interquartile ranges.

Second, internal consistency testing was conducted for the 12 core indicators of occupational physical fitness and their three dimensions. Cronbach’s α was used to evaluate the consistency of the preliminary conceptual framework across different groups. Because the enterprise sample size was small, Cronbach’s α for the enterprise group was used only as an exploratory reference and not as reliability evidence for a mature scale. Expert content validity was evaluated using the I-CVI and S-CVI/Ave. The I-CVI was calculated as the proportion of experts who rated each indicator as 3 or 4 among the total number of experts, while the S-CVI/Ave was calculated as the mean of the I-CVI values across all indicators. For multiple-response items, each response option was summarized using the number and percentage of eligible respondents who selected that option. The denominator was the number of respondents eligible to answer the corresponding item rather than the total number of selections; therefore, percentages across response options could sum to more than 100%. Because questionnaires containing unanswered items were excluded during data screening, there were no missing responses for the multiple-response items reported in the final analytical sample. For the student multiple-response items reported in the main text and figures (QB9–QB11), the denominator was 1,863 students. For teacher item QA7a, which was administered only to teachers whose main teaching field was physical education or physical fitness-related courses, the denominator was 17 eligible teachers; other reported teacher multiple-response items used the 37-teacher sample as the denominator. Enterprise multiple-response items used all 24 enterprise respondents as the denominator. For QB9, “None” was intended to indicate the absence of the listed physical-discomfort symptoms. Because the survey platform did not technically enforce mutual exclusivity, 18 respondents selected “None” together with at least one specific symptom. For logical consistency, the specific symptom selections were retained and the contradictory “None” selection was recoded as unselected; no respondent was excluded on this basis.

Third, between-group comparisons were conducted for the occupational physical fitness dimension scores and core indicator scores among students, teachers, and enterprise personnel. Given the ordinal five-point rating scale, the pronounced imbalance in stakeholder-group sample sizes, and the exploratory purpose of the cross-stakeholder analysis, the Kruskal–Wallis H test was used as a rank-based omnibus procedure to compare rating distributions among the three groups. Although the Kruskal–Wallis test does not require equal group sizes, the markedly smaller teacher and enterprise samples limit the precision and statistical power of these comparisons. Accordingly, the analyses were treated as exploratory and were not interpreted as confirmatory tests of population-level stakeholder differences. Because the Kruskal–Wallis procedure may reflect differences in distributional shape as well as location, its results were interpreted as exploratory evidence of differences in rank distributions rather than specifically as tests of equality of group medians. For omnibus comparisons with a nominal raw p < 0.05, Bonferroni-adjusted pairwise comparisons were additionally calculated for exploratory purposes. However, pairwise findings were interpreted as multiplicity-controlled evidence only when the corresponding omnibus comparison remained statistically supported after FDR correction across the full family of outcomes. Because the exploratory study did not prespecify a single primary outcome, the three dimension-level analyses were regarded as higher-level framework comparisons, whereas the 12 individual-indicator analyses were treated as secondary exploratory comparisons. To address multiplicity, the Benjamini–Hochberg false discovery rate procedure was applied jointly to the p values from all 15 omnibus Kruskal–Wallis tests [24]. Both raw p values and FDR-adjusted q values are reported. Statistical support after multiplicity correction was defined as q < 0.05.

To examine potential ceiling effects and limited response discrimination in the five-point importance ratings, the full response distributions for scores 1–5 were additionally tabulated for each of the 12 core indicators within each stakeholder group. The proportions selecting the maximum response category (5, “Very important”) and the two upper response categories (4–5) were examined descriptively as indicators of upper-end response concentration. Because no formal ceiling-effect criterion had been prespecified for this exploratory instrument, these distributions were used to characterize possible ceiling effects rather than to apply a post hoc binary classification of individual items.

For between-group comparisons, test statistics, p values, and effect sizes were reported simultaneously to avoid interpreting the results solely on the basis of statistical significance and to enhance the practical interpretation of the findings [25]. Cross-stakeholder comparisons were restricted to the 12 common importance-rating items and the three dimension scores derived from these common items. Stakeholder-specific questions that differed in wording, content, or response format across questionnaire versions were analyzed descriptively within the relevant stakeholder group and were not subjected to cross-group statistical testing. Because recruitment occurred partly through class-, institution-, and enterprise-based channels, responses from participants within the same recruitment unit may not have been fully independent. Analyses were conducted at the individual-participant level without adjustment for clustering. Because college-, class-, and enterprise-level cluster identifiers were not retained in the analytical dataset, intraclass correlation coefficients could not be estimated, and multilevel, generalized estimating equation, or cluster-robust analyses could not be conducted reliably. Accordingly, the between-group comparisons were interpreted as exploratory, with greater emphasis placed on effect sizes and descriptive patterns than on statistical significance alone. A nonsignificant Kruskal–Wallis result was interpreted only as an absence of a statistically detected between-group difference in the present dataset and not as evidence of equivalence, agreement, or cross-stakeholder consensus. No equivalence-testing procedure was conducted because equivalence margins were not prespecified.

Finally, interview data were processed using thematic analysis. Two researchers independently read the interview texts and conducted initial coding. They then compared the coding results, merged similar codes, and generated themes. Discrepancies in coding were resolved through discussion until consensus was reached. The qualitative results were not used for statistical inference; rather, they were used to explain the phenomena identified in the questionnaire survey and, together with the quantitative results, to support the exploratory construction of the preliminary conceptual framework of occupational physical fitness qualities. It should be noted that this study did not conduct exploratory factor analysis, confirmatory factor analysis, or structural equation modeling. Therefore, the proposed framework should be regarded as a preliminary theoretical model based on multiple sources of evidence and requires further validation in future research.

To improve data-processing transparency and reproducibility, detailed documentation of the final analytical dataset is provided in the Supporting Information. S2 Table provides the complete data dictionary, including exact variable names and labels, response codes, identification of multiple-response variables, structural missing-data conventions, derived-score formulas, data-screening and exclusion rules, and documented data-processing decisions. Because the original quantitative analyses were conducted primarily through the SPSS graphical user interface, a complete original syntax file was not retained. Accordingly, S1 File provides reconstructed and verified reproducible SPSS syntax prepared during revision to reproduce the final reported questionnaire-based quantitative analyses. S2 File provides the data-processing, de-identification, and reproducibility README. Detailed operational definitions of the screening criteria, the available participant-disposition information, and the limitations of the historical pre-cleaning records are provided in S2 Table and S2 File. The publicly shared dataset corresponds to the final screened analytical sample (n = 1,924), comprising 1,863 students, 37 teachers, and 24 enterprise personnel. Before public release, the dataset was specifically reviewed for potential indirect-identification risk, with particular attention to combinations of background characteristics in the small teacher and enterprise personnel groups. All questionnaire free-text response fields were removed from the public-release dataset following this disclosure-risk review because such responses could contain potentially identifying contextual information. These de-identification procedures did not alter the final analytical sample size or any quantitative analysis reported in the manuscript.

### 2.11. Use of artificial intelligence tools and technologies

ChatGPT (GPT-5.6 Sol, OpenAI) was used to assist in generating graphical elements for Figs 4 and 5. The AI-assisted outputs were reviewed and edited by the authors, and all scientific content, figure structure, and interpretations were determined and verified by the authors.

## 3. Results

### 3.1. Basic characteristics of the sample

Of the 2,106 questionnaires returned, 182 were excluded during data screening and 1,924 were retained in the final analytical sample, comprising 1,863 students, 37 teachers, and 24 enterprise personnel. The student sample covered students from the first to the fourth year as well as those in the pre-graduation internship stage. Their major fields included computer technology, network technology, cloud computing, and other information technology-related programs. The teacher sample included information technology teachers and physical education teachers. The enterprise sample was mainly drawn from software development, cybersecurity, artificial intelligence, and other information technology-related industries. Overall, students accounted for the majority of the sample, whereas the numbers of teachers and enterprise personnel were relatively small. Therefore, findings from the teacher and enterprise groups were interpreted primarily as descriptive and exploratory evidence. Detailed demographic, educational, and professional characteristics of the three survey groups are presented in Table 2.

**Table 2:** Demographic, educational, and professional characteristics of survey participants.

Participant group	Characteristic	Category	n (%)
Students (n = 1,863)	Gender	Male	840 (45.09)
		Female	1,023 (54.91)
	Year/stage of study	First year	824 (44.23)
		Second year	717 (38.49)
		Third year	274 (14.71)
		Fourth year / internship stage	15 (0.81)
		Pre-graduation / awaiting formal graduation certificate^a^	33 (1.77)
	Information technology field	Software development and engineering	50 (2.68)
		Communication-related fields	486 (26.09)
		Network technology	318 (17.07)
		Cloud computing technology and applications	328 (17.61)
		Blockchain technology	86 (4.62)
		Big data and artificial intelligence	31 (1.66)
		Internet of Things technology	35 (1.88)
		Digital media technology	29 (1.56)
		Information technology operation and maintenance	19 (1.02)
		Other information technology-related fields	481 (25.82)
	Weekly professional practical-operation time	<10 h/week	1,183 (63.50)
		10–20 h/week	481 (25.82)
		20–30 h/week	102 (5.48)
		>30 h/week	97 (5.21)
	Information technology-related internship experience	Yes	161 (8.64)
		No	1,702 (91.36)
	Weekly exercise duration	Almost no exercise	275 (14.76)
		1–3 h/week	969 (52.01)
		3–5 h/week	349 (18.73)
		5–7 h/week	102 (5.48)
		>7 h/week	168 (9.02)
Teachers (n = 37)	Gender	Male	12 (32.43)
		Female	25 (67.57)
	Age group	20–30 years	15 (40.54)
		31–40 years	11 (29.73)
		41–50 years	10 (27.03)
		>50 years	1 (2.70)
	Institution type	General undergraduate institution	9 (24.32)
		Vocational undergraduate institution	2 (5.41)
		Higher vocational college / junior college	26 (70.27)
	Teaching experience	1–3 years	16 (43.24)
		4–6 years	7 (18.92)
		7–10 years	1 (2.70)
		>10 years	13 (35.14)
	Professional title	Teaching assistant	19 (51.35)
		Lecturer	10 (27.03)
		Associate professor	4 (10.81)
		Professor	4 (10.81)
	Main teaching field	Basic professional courses	11 (29.73)
		Core professional courses	6 (16.22)
		General education courses	3 (8.11)
		Physical education courses	17 (45.95)
Enterprise personnel (n = 24)	Industry sector	Software development and services	11 (45.83)
		Network and information security	3 (12.50)
		Internet of Things and smart devices	0 (0.00)
		Artificial intelligence and big data	3 (12.50)
		Digital media and design	2 (8.33)
		Information technology operation and maintenance services	4 (16.67)
		Other	1 (4.17)
	Enterprise size	≤50 employees	7 (29.17)
		51–200 employees	7 (29.17)
		201–500 employees	2 (8.33)
		≥501 employees	8 (33.33)
	Vocational-college IT graduates recruited in the past 3 years	≤5	9 (37.50)
		6–15	1 (4.17)
		16–30	7 (29.17)
		≥31	7 (29.17)
	Main area of responsibility	Human resources recruitment and training	7 (29.17)
		Technical department management	0 (0.00)
		Front-line technical R&D / operation and maintenance	13 (54.17)
		Other	4 (16.67)

Percentages were calculated within each stakeholder group and may not total 100% because of rounding.

^a^“Pre-graduation / awaiting formal graduation certificate” refers to students who had completed their required coursework and were awaiting formal issuance of their graduation certificates; they had not formally graduated at the time of participation.

^b^Teachers were eligible based on the relevance of their teaching or research activities to vocational education or the training of information technology students, irrespective of institution type.

^c^Only background variables prospectively collected in the original questionnaires are reported. Student age and specific institution identity were not collected; individual demographic characteristics such as age and gender were not collected in the enterprise questionnaire.

### 3.2. Occupational physical fitness-related discomfort and training status among students

Among the student participants, 68.71% selected either “Frequently” or “Occasionally” when asked whether they experienced physical discomfort after professional practical operations, such as long-term computer use or equipment debugging. These response categories did not have predefined numerical frequency thresholds or a fixed retrospective recall period and should therefore be interpreted as descriptive self-reported frequency categories rather than as a period-prevalence estimate. The most frequently selected symptom category was dry eyes or blurred vision (44.50%), followed by cervical or lumbar soreness (38.06%) and physical fatigue or insufficient endurance (25.93%). The distribution of self-reported physical-discomfort categories among students is shown in Fig 1.

**Fig 1:**
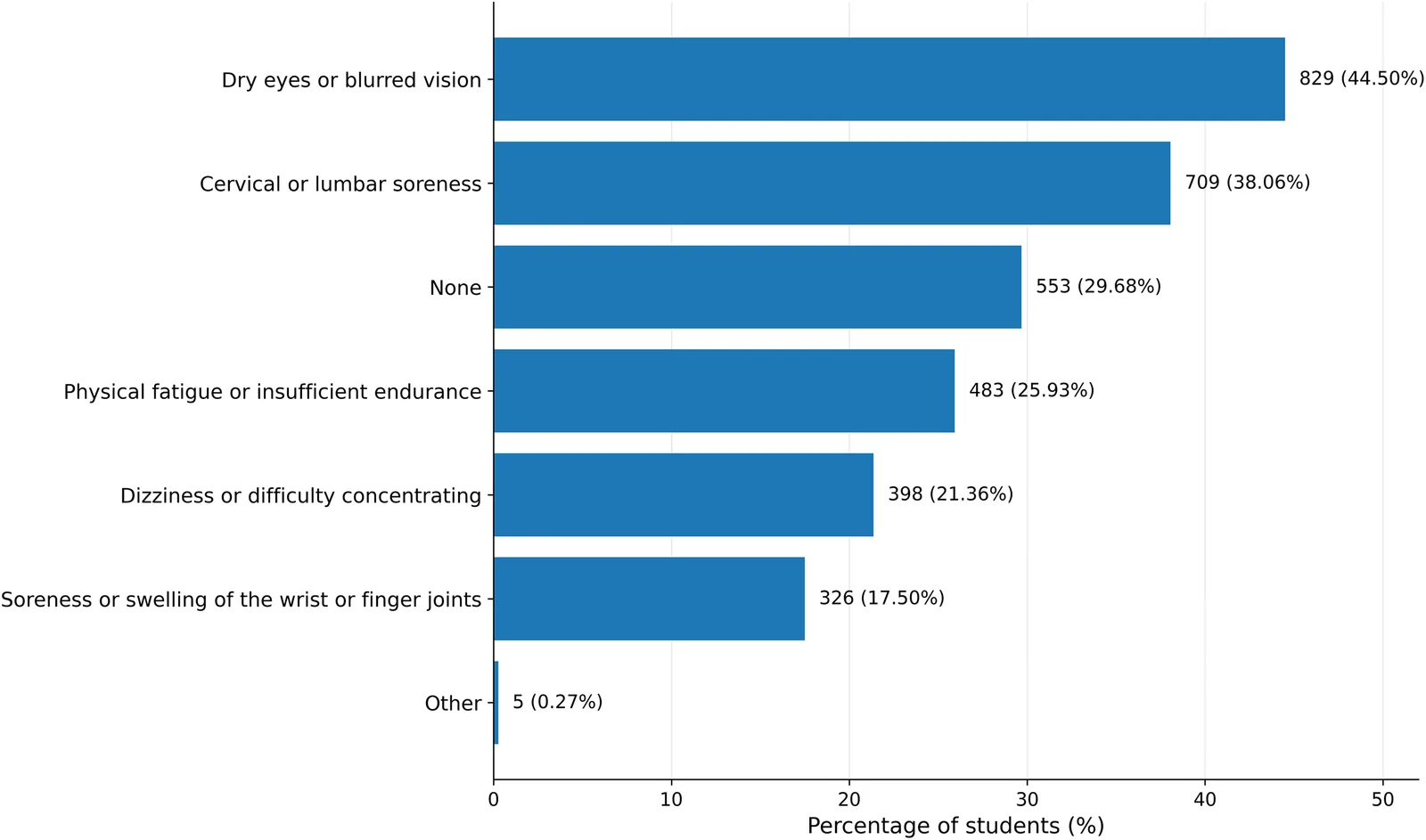
Distribution of self-reported physical-discomfort categories among students. QB9 was a multiple-response item. Percentages were calculated using all 1,863 student respondents as the denominator and may sum to more than 100%. There were no missing responses. For 18 respondents who selected “None” together with at least one specific symptom, the specific symptom selection(s) were retained and “None” was recoded as unselected. “Cervical or lumbar soreness” represents a single combined response option in QB9; cervical and lumbar symptoms were not assessed separately.

In terms of physical exercise, 14.76% of students selected “Almost no exercise,” 52.01% reported 1–3 h/week, 18.73% reported 3–5 h/week, 5.48% reported 5–7 h/week, and 9.02% reported >7 h/week. Because the questionnaire assessed weekly exercise duration without measuring intensity or frequency, these categories were not interpreted as indicating whether students met or failed to meet an established physical-activity recommendation. Regarding exercise content, students mainly participated in general forms of exercise such as running and ball games. The qualitative interviews with students and teachers further indicated that participation in occupation-specific physical fitness training, including visual relaxation, musculoskeletal relaxation, postural adjustment, and sedentary behavior interruption, was very limited in current educational practice. Taken together, the survey and interview findings suggest that students’ exercise patterns were predominantly centered on general physical activity, while occupation-specific physical fitness training relevant to information technology learning and future work was only limitedly incorporated into current practice.

### 3.3. Self-reported psychological experiences and coping strategies among students

For the multiple-response item concerning psychological experiences during professional learning or practice, 26.89% of students selected “Frequently feeling nervous because of concerns about employment or internship performance,” and 22.38% selected “Feeling so anxious before exams or project deadlines that your sleep is affected.” The item did not specify a uniform retrospective recall period and was not based on a validated clinical or symptom-specific instrument; therefore, these percentages represent response-option endorsement proportions rather than prevalence estimates or clinical assessments of anxiety, insomnia, sleep disturbance, or other mental health conditions. The distribution of self-reported psychological experiences among students is shown in Fig 2.

**Fig 2:**
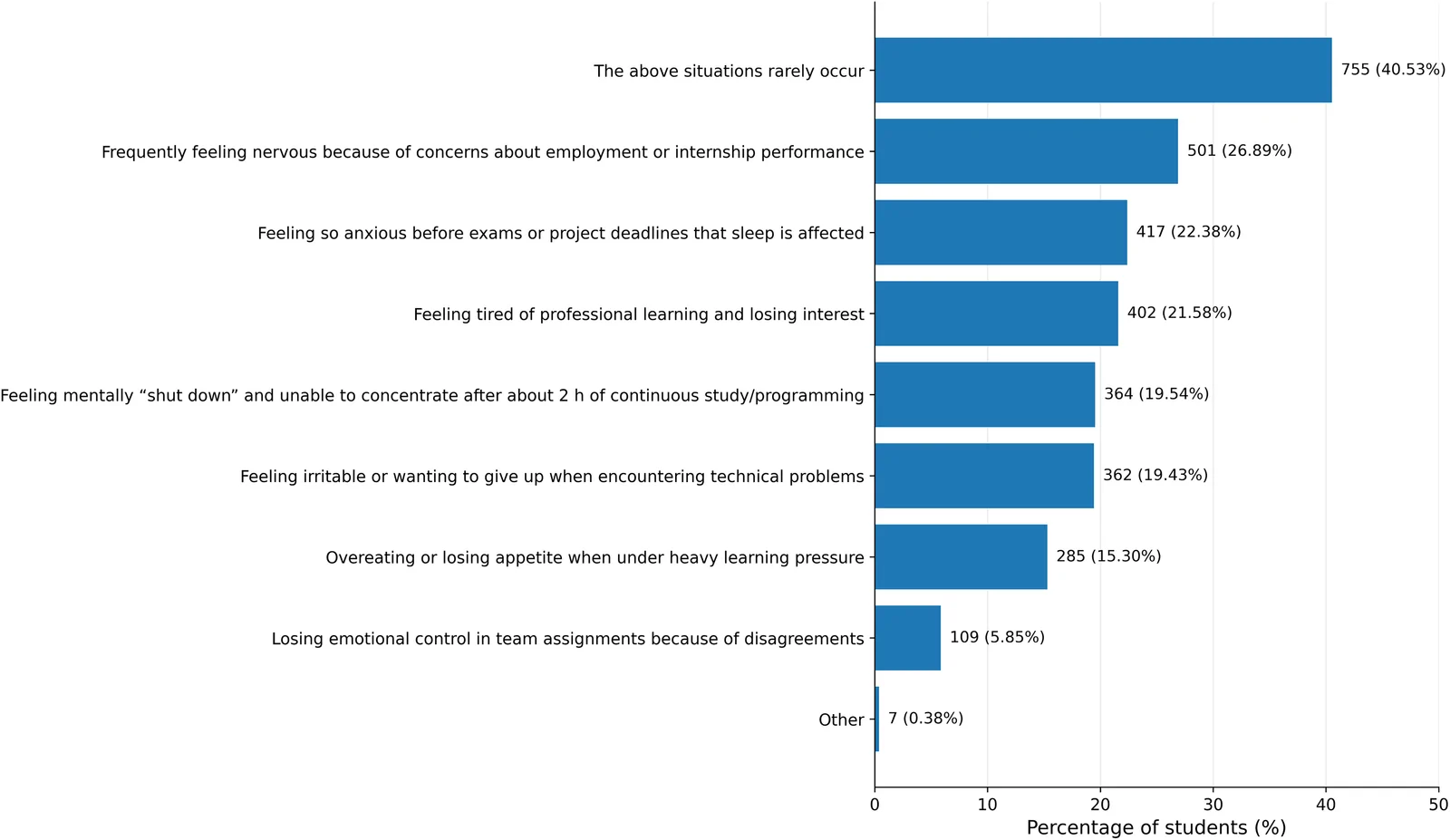
Distribution of self-reported psychological experiences among students. QB10 was a multiple-response item. Percentages were calculated using all 1,863 student respondents as the denominator; there were no missing responses, and percentages may therefore sum to more than 100%. The reported values represent the proportions of respondents selecting each listed experience rather than period-prevalence estimates or clinical diagnoses.

Regarding coping strategies, 1,269/1,863 students (68.12%) selected listening to music or watching videos, 859 (46.11%) selected talking with friends or family members, and 778 (41.76%) selected exercise or physical activity. The distribution of coping strategies reported by students is shown in Fig 3.

**Fig 3:**
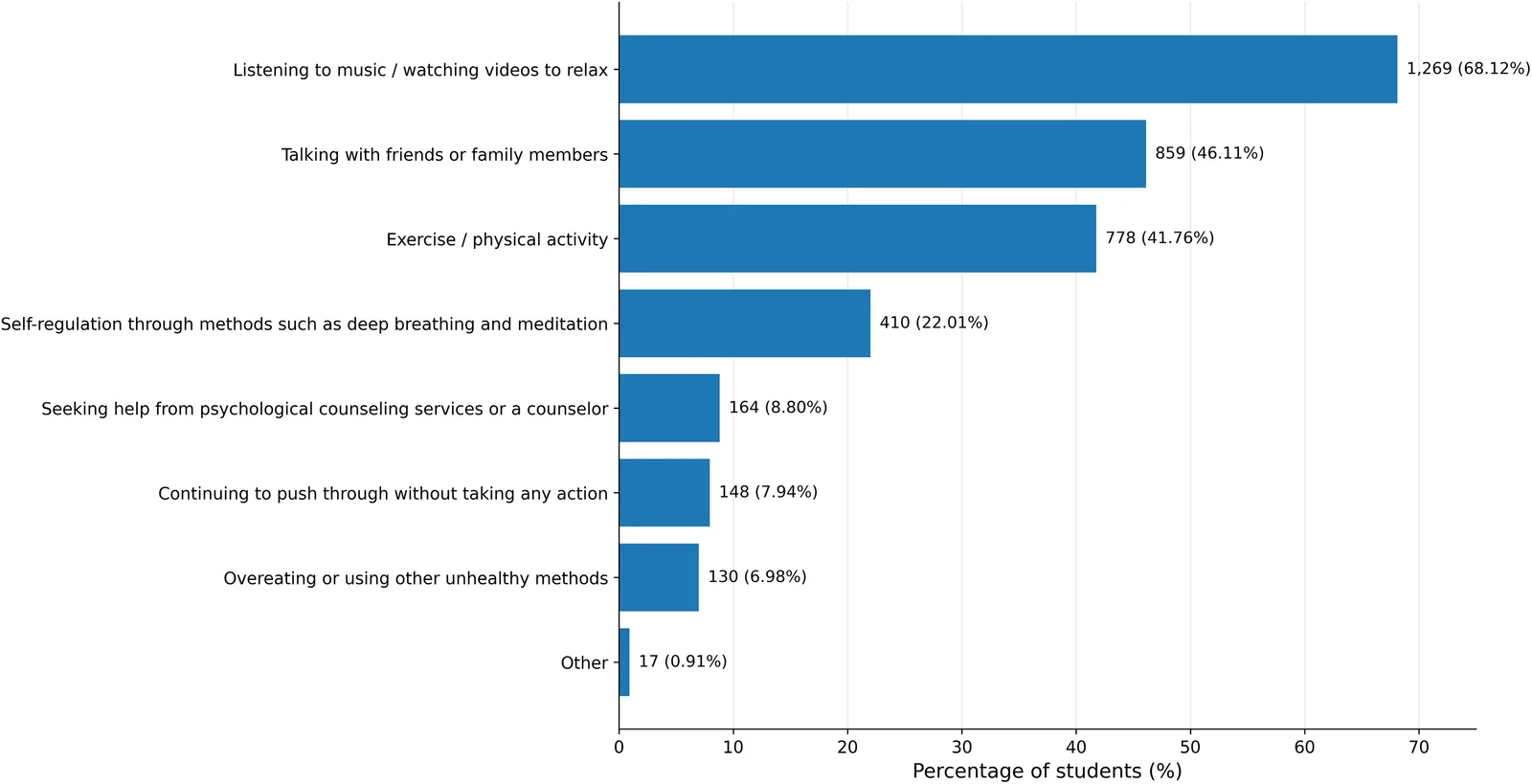
Distribution of coping strategies reported by students. QB11 was a multiple-response item. Percentages were calculated using all 1,863 student respondents as the denominator; there were no missing responses, and percentages may therefore sum to more than 100%. The reported values represent the proportions of respondents selecting each listed coping strategy.

### 3.4. Occupational physical fitness training provision reported by surveyed teachers

Among the 17 teachers whose main teaching field was physical education and who were therefore eligible to answer QA7a, 13 (76.47%) selected a lack of specific data on the physical fitness demands of information technology positions, 11 (64.71%) selected low student interest in courses integrating professional learning and physical fitness, and 10 (58.82%) selected difficulty matching existing physical fitness training methods to professional job scenarios. Because QA7a allowed multiple responses, percentages were calculated using the 17 eligible teachers as the denominator and may sum to more than 100%.

Among all 37 teachers, 29 (78.38%) selected physical fitness training equipment, 28 (75.68%) selected support from professional physical fitness coaches, and 22 (59.46%) selected specialized occupational physical fitness training materials as needed teaching resources. In addition, 27/37 teachers (72.97%) indicated that enterprises should provide standards for job-related physical fitness demands. Among the 17 teachers eligible for the enterprise-investigation item, 15 (88.24%) indicated that they were willing or very willing to participate in on-site investigations of enterprise information technology positions.

### 3.5. Enterprise perceptions of and participation in occupational physical fitness

Among the 24 enterprise respondents, 13 (54.17%) selected decreased work efficiency, 13 (54.17%) selected an increased frequency of taking leave, and 8 (33.33%) selected an increased rate of operational errors in the multiple-response item concerning perceived problems associated with physical fitness. Because multiple selections were permitted, each percentage used all 24 enterprise respondents as the denominator and the percentages were not mutually exclusive.

In the recruitment-related item, 5/24 respondents (20.83%) reported paying very close attention and 10/24 (41.67%) reported paying some attention to candidates’ occupational physical fitness-related performance, corresponding to 15/24 (62.50%) across these two response categories.

When asked what forms of support enterprises could provide to vocational colleges (QC13; multiple responses), 10/24 respondents (41.67%) selected providing standards for job-related physical fitness demands, 10/24 (41.67%) selected participation in physical fitness curriculum design, and 12/24 (50.00%) selected providing physical fitness training scenarios during enterprise practice.

### 3.6. Internal consistency and content validity of the occupational physical fitness indicators

This study conducted internal consistency testing on the importance ratings of the 12 core indicators of occupational physical fitness. Cronbach’s α was 0.987 for the student group, 0.970 for the teacher group, and 0.979 for the enterprise group. These coefficients describe internal consistency only and should not be interpreted as evidence of factorial or structural validity. The corresponding dimension-specific Cronbach’s α coefficients are presented in Table 3. Given the small enterprise sample (n = 24), the enterprise-group coefficients are reported only as sample-specific exploratory estimates and should not be interpreted as stable population-level reliability coefficients or as evidence of psychometric validation.

**Table 3:** Internal consistency analysis of the core indicators of occupational physical fitness.

Group	N	Overall Cronbach’s α	General Physical Fitness α	Occupation-Specific Physical Fitness α	Psychological Fitness α
Students	1863	0.987	0.966	0.975	0.979
Teachers	37	0.970	0.910	0.937	0.973
Enterprise personnel	24	0.979	0.952	0.979	0.980

Because the enterprise sample was small (n = 24), its Cronbach’s α coefficients are presented for exploratory descriptive purposes only and should be interpreted cautiously.

For the expert content evaluation, the I-CVI values for the 12 indicators ranged from 0.88 to 1.00 across the evaluated criteria, and the S-CVI/Ave was 0.95. Expert comments primarily concerned the accuracy of indicator names and item wording. Based on these comments, “visual tolerance” was revised to “visual load tolerance,” and the wording of several items was simplified. The results of the expert content validity evaluation are presented in Table 4.

**Table 4:** Expert content validity evaluation results for the occupational physical fitness quality indicators of information technology programs.

Dimension	Indicator	Relevance I-CVI	Clarity I-CVI	Applicability I-CVI	Dimensional Assignment I-CVI	Decision
General physical fitness	Cardiorespiratory endurance (A1)	1	1	0.88	1	Retained
	Muscular strength (A2)	1	0.88	0.88	1	Retained
	Flexibility (A3)	0.88	1	0.88	1	Retained
	Bodily coordination (A4)	0.88	0.88	0.88	0.88	Retained
Occupation-specific physical fitness	Sedentary tolerance (B1)	1	1	1	1	Retained
	Visual load tolerance (B2)	1	0.88	1	1	Retained after wording revision
	Fine motor operation endurance (B3)	0.88	0.88	0.88	0.88	Retained
	Static postural endurance (B4)	1	0.88	1	1	Retained
Psychological fitness	Stress resistance (C1)	1	1	1	1	Retained
	Sustained concentration (C2)	1	1	1	1	Retained
	Emotional regulation (C3)	0.88	1	0.88	1	Retained
	Emergency response capacity (C4)	0.88	0.88	0.88	0.88	Retained after wording revision

The I-CVI values are direct proportions based on eight expert ratings; therefore, 0.88 represents 7/8 experts and 1.00 represents 8/8 experts rating an indicator as 3 or 4. These values should be interpreted as preliminary panel-specific evidence of expert agreement rather than as precise population-level estimates of content validity.

### 3.7. Exploratory comparison of perceived occupational physical fitness priorities among students, teachers, and enterprise personnel

Overall, ratings for the three dimensions and most core indicators were concentrated toward the upper end of the five-point importance scale across the three stakeholder groups. The results are shown in Table 5.

**Table 5:** Exploratory cross-stakeholder comparison of importance ratings for occupational physical fitness dimensions and core indicators.

Dimension/Indicator	Students Mdn (IQR) n = 1,863	Teachers Mdn (IQR) n = 37	Enterprise personnel Mdn (IQR) n = 24	H value	Raw p value	FDR-adjusted q value	ε²
**General physical fitness**	4.00 (2.00)	4.00 (1.25)	4.00 (2.00)	0.011	0.9943	0.9943	0
**Occupation-specific physical fitness**	4.00 (2.00)	4.25 (1.00)	4.00 (2.00)	3.133	0.2087	0.5502	0.001
**Psychological fitness**	4.00 (2.00)	4.25 (1.00)	4.625 (1.25)	4.579	0.1013	0.381	0.001
Cardiorespiratory endurance (A1)	4.00 (2.00)	4.00 (1.00)	4.00 (2.00)	1.37	0.5041	0.5817	0
Muscular strength (A2)	4.00 (2.00)	4.00 (1.00)	4.00 (2.00)	1.463	0.4811	0.5817	0
Flexibility (A3)	4.00 (2.00)	4.00 (2.00)	4.00 (2.00)	2.285	0.319	0.5502	0
Bodily coordination (A4)	4.00 (2.00)	4.00 (2.00)	4.00 (1.25)	0.591	0.7442	0.7974	0
Sedentary tolerance (B1)	4.00 (2.00)	4.00 (1.00)	4.00 (2.00)	4.573	0.1016	0.381	0.001
Visual load tolerance (B2)	4.00 (2.00)	4.00 (1.00)	4.00 (2.00)	2.876	0.2374	0.5502	0
Fine motor operation endurance (B3)	4.00 (2.00)	4.00 (1.00)	4.00 (2.00)	2.04	0.3606	0.5502	0
Static postural endurance (B4)	4.00 (2.00)	4.00 (1.00)	4.00 (2.00)	1.511	0.4698	0.5817	0
Stress resistance (C1)	4.00 (2.00)	5.00 (1.00)	5.00 (1.25)	4.816	0.09	0.381	0.001
Sustained concentration (C2)	4.00 (2.00)	5.00 (1.00)	5.00 (1.25)	8.574	0.0137	**0.2055**	0.003
Emotional regulation (C3)	4.00 (2.00)	4.00 (1.00)	4.50 (1.25)	2.474	0.2903	0.5502	0
Emergency response capacity (C4)	4.00 (2.00)	4.00 (1.00)	5.00 (2.00)	2.006	0.3668	0.5502	0

Student group, n = 1,863; teacher group, n = 37; enterprise personnel group, n = 24. Mdn = median; IQR = interquartile range; ε² = effect size for the Kruskal–Wallis H test. Raw p values are reported together with Benjamini–Hochberg false-discovery-rate (FDR)-adjusted q values. The FDR procedure was applied jointly across all 15 omnibus Kruskal–Wallis comparisons, comprising three dimension-level comparisons and 12 indicator-level comparisons. Statistical support after multiplicity correction was defined as q < 0.05. None of the 15 comparisons met this criterion; therefore, no post hoc pairwise comparison was interpreted as statistically supported. The 12 core indicators were administered with identical indicator names, descriptions, response categories, coding direction, and dimensional composition across the three questionnaire versions, although the introductory question stem was contextually adapted for each stakeholder group. Accordingly, these analyses should be interpreted as exploratory cross-stakeholder comparisons rather than as evidence of formal measurement invariance, statistical equivalence, psychometric equivalence, consistency, or cross-stakeholder consensus.

For transparency, the Bonferroni-adjusted pairwise result associated with the nominally significant unadjusted C2 omnibus test is retained in the table; however, because the corresponding omnibus result did not remain statistically supported after FDR correction (q = 0.2055), the pairwise result is not interpreted as multiplicity-controlled evidence of a stakeholder difference.

The exploratory Kruskal–Wallis analyses did not identify any dimension-level or indicator-level comparison with an FDR-adjusted q value below 0.05. Sustained concentration (C2) yielded the smallest raw p value among the 15 comparisons (H[2] = 8.574, raw p = 0.0137, ε² = 0.003), but the corresponding FDR-adjusted value was q = 0.2055. A Bonferroni-adjusted student–teacher pairwise comparison yielded p = 0.025; however, because the C2 omnibus comparison did not remain supported after correction across the full family of outcomes, this pairwise result is reported for transparency only and is not interpreted as robust evidence of a stakeholder difference. Overall, none of the 15 omnibus comparisons met the prespecified q < 0.05 criterion.

Inspection of the full five-point response distributions showed concentration toward the upper response categories across the 12 indicators. Among students, 34.03%–39.35% selected rating 5 and 68.17%–71.44% selected ratings 4–5. Among teachers, the corresponding ranges were 27.03%–54.05% and 56.76%–94.59%, respectively. Among enterprise personnel, the corresponding ranges were 33.33%–54.17% and 62.50%–75.00%, respectively. Full response distributions are presented in S5 Table.

Before multiplicity adjustment, C2 yielded the smallest raw p value among the 15 comparisons (H[2] = 8.574, raw p = 0.0137, ε² = 0.003). After Benjamini–Hochberg FDR correction, q = 0.2055. No dimension-level or indicator-level comparison had an FDR-adjusted q value below 0.05.

Further integration of the quantitative and qualitative findings identified three complementary but conceptually distinct domains. At the student level, the survey documented self-reported physical and psychological experiences, exercise and training participation, and barriers to occupation-specific training. At the stakeholder level, students, teachers, and enterprise personnel provided importance ratings reflecting perceived priorities among the proposed occupational physical fitness dimensions and indicators. At the provision level, teachers and enterprise personnel reported constraints related to job-demand information, specialized teaching resources, curriculum provision, and school–enterprise participation. These three domains were retained as conceptually distinct components in the mixed-methods integration rather than being treated as interchangeable measures of a single unmet need. The integration of these findings is summarized in Fig 4.

**Fig 4:**
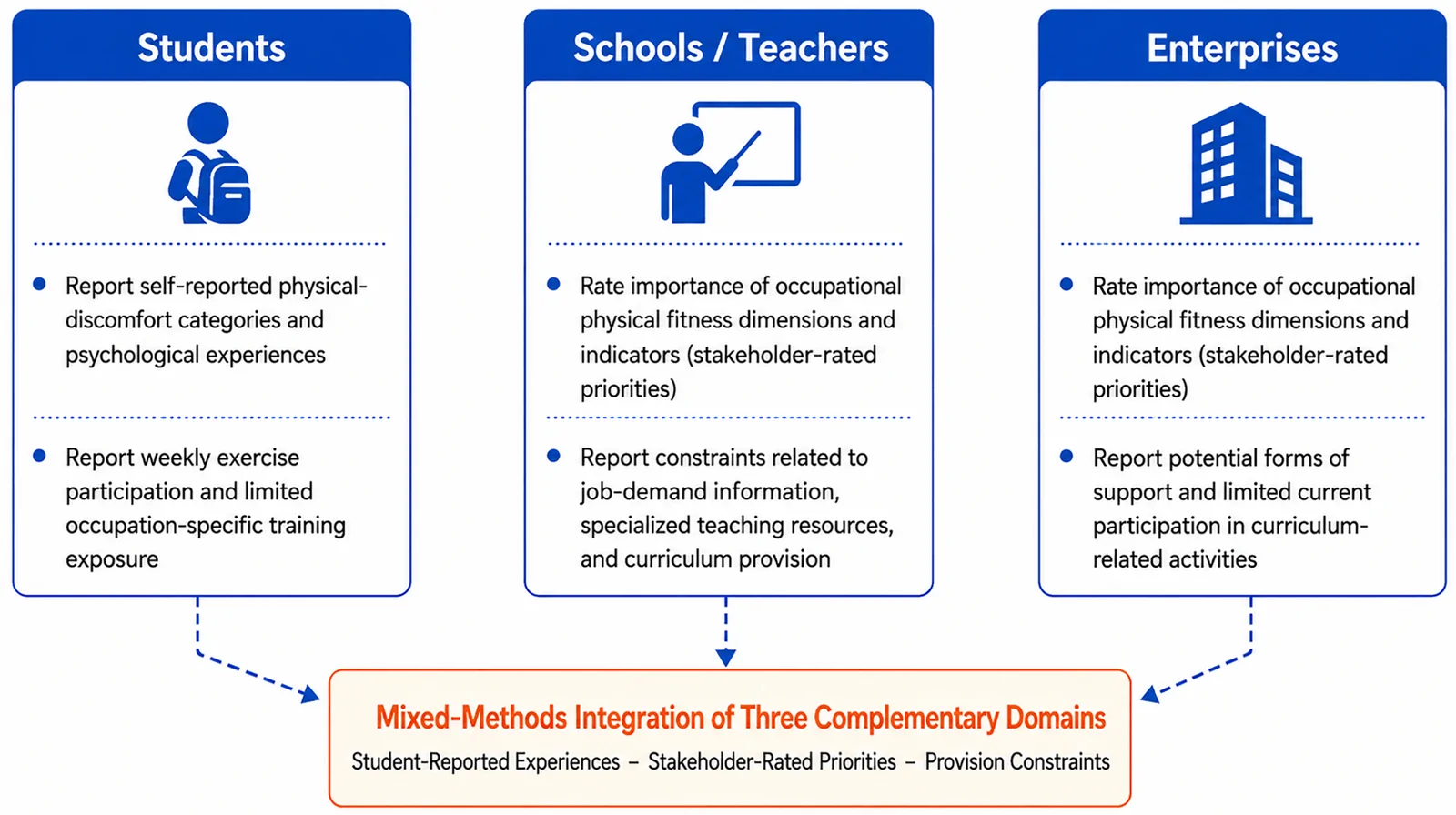
Mixed-methods integration of student-reported experiences, stakeholder-rated priorities, and provision constraints related to occupational physical fitness (Graphical elements in this figure were generated with the assistance of ChatGPT (GPT-5.6 Sol, OpenAI) and subsequently reviewed, edited, and arranged by the authors).

### 3.8. Construction of a preliminary conceptual framework of occupational physical fitness

Based on the integration of the literature-derived indicator structure, expert content evaluation, questionnaire findings, and interview themes, a preliminary conceptual framework comprising three dimensions and 12 core indicators was constructed. General physical fitness comprised cardiorespiratory endurance, muscular strength, flexibility, and bodily coordination; occupation-specific physical fitness comprised sedentary tolerance, visual load tolerance, fine motor operation endurance, and static postural endurance; and psychological fitness comprised stress resistance, sustained concentration, emotional regulation, and emergency response capacity. The resulting framework is presented in Fig 5.

**Fig 5:**
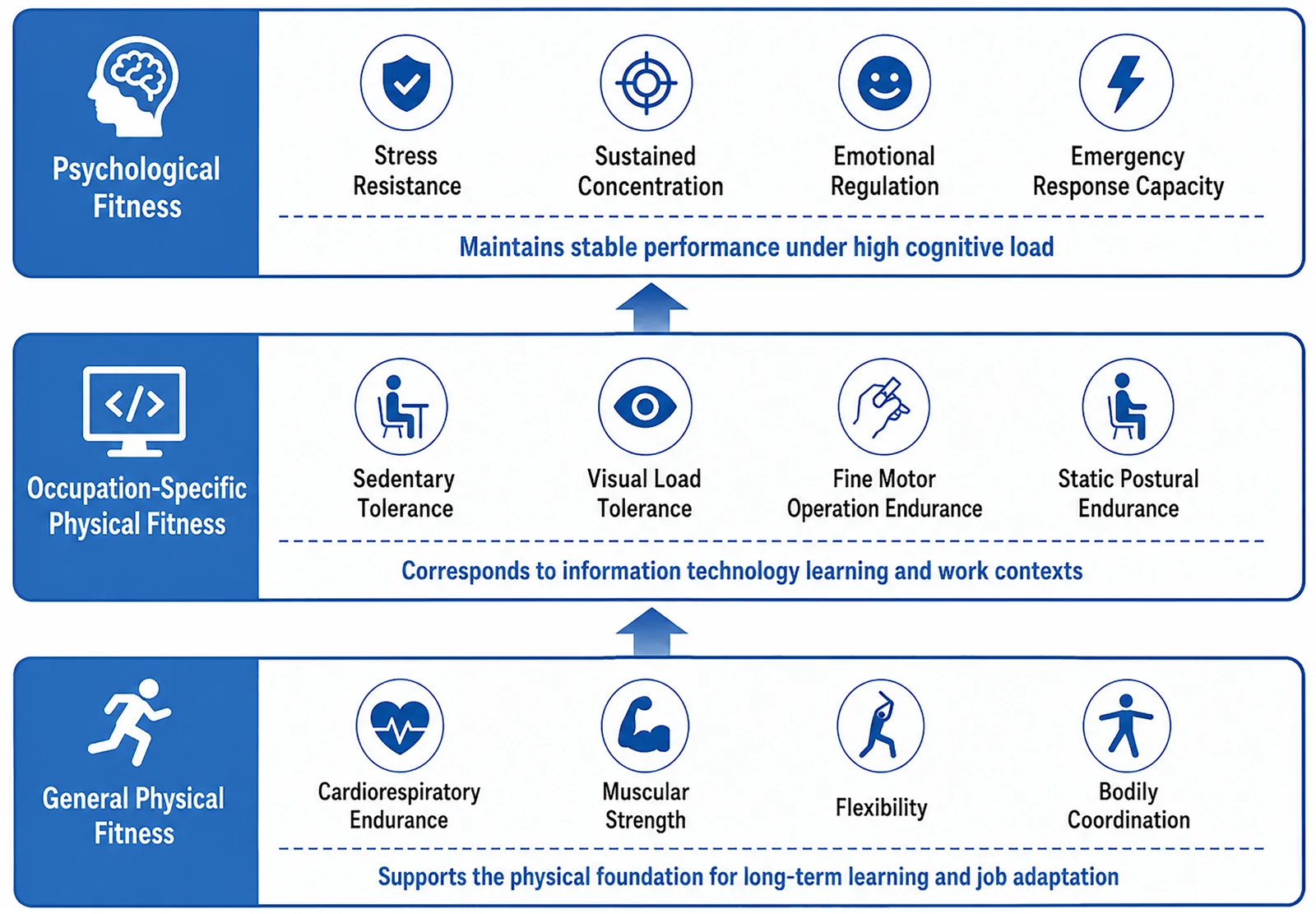
Preliminary conceptual framework of occupational physical fitness qualities for information technology programs (Graphical elements in this figure were generated with the assistance of ChatGPT (GPT-5.6 Sol, OpenAI) and subsequently reviewed, edited, and arranged by the authors).

## 4. Discussion

This study examined occupational physical fitness-related conditions and perceived priorities among information technology students from selected vocational colleges in Shanxi Province, China, with supplementary perspectives from teachers and enterprise personnel. Based on literature analysis, expert content validity evaluation, questionnaire survey data, and interview data, this study further proposed a preliminary conceptual framework. The main findings were as follows.

First, students commonly reported specific physical-discomfort categories, including dry eyes or blurred vision, cervical or lumbar soreness, and physical fatigue or insufficient endurance, together with several self-reported psychological experiences related to professional learning, project deadlines, and employment or internship concerns. Weekly exercise duration was most commonly reported in the 1–3 h/week category, while occupation-specific training remained relatively limited in the educational context examined. The pattern of self-reported physical discomfort observed in the present sample is broadly compatible with previous research on prolonged computer and digital-device use. Previous studies have associated sustained digital-device exposure with digital eye strain, while prolonged computer work, postural load, workstation characteristics, and extended sitting have also been associated with neck and lower-back discomfort [1,2,10]. These findings provide relevant occupational-health context for the physical-discomfort categories reported by participants in the present study. However, because the current study was cross-sectional and relied on self-reported questionnaire responses, no causal relationship between these occupational or learning-related exposures and the reported discomfort can be inferred.

The self-reported psychological experiences observed in the present sample should be interpreted in the context of professional learning, project-based tasks, and preparation for internships or employment rather than as clinically assessed mental-health outcomes. Previous studies of information technology workers have suggested that work deadlines, role stress, sustained technical tasks, and organizational demands may be associated with occupational stress, mental-health symptoms, and burnout [3,8,15]. These findings provide relevant contextual evidence for understanding the experiences reported by students in the present study. More broadly, stress and sleep disturbances are closely interrelated among university students, while digital-device exposure near the sleep period may additionally affect sleep and circadian regulation [26,27]. Nevertheless, the present study did not assess sleep architecture, timing of screen exposure, circadian or neuroendocrine biomarkers, or clinical mental-health outcomes using validated instruments; these mechanisms should therefore be regarded as hypotheses for future investigation rather than as explanations established by the present cross-sectional data.

The coping responses reported by students provide additional context for understanding how they managed psychologically demanding experiences. Listening to music or watching videos was the most frequently selected strategy, followed by talking with friends or family members and engaging in exercise or physical activity. By contrast, structured approaches such as breathing or meditation and psychological counseling were selected less frequently. These descriptive patterns suggest that structured self-regulation strategies were not among the most commonly reported coping approaches in the present sample. In the context of information technology education, these findings may support further investigation of whether educational approaches addressing stress regulation, sustained concentration, and emotional regulation could be feasibly incorporated into occupation-related health and physical education programs [28]. However, the present cross-sectional findings do not establish the effectiveness or necessity of any specific psychological training intervention.

Second, the teacher findings highlighted perceived constraints related to limited access to job-demand information, difficulty matching physical fitness training methods to occupational scenarios, and the availability of specialized teaching resources and professional support. The relatively frequent selection of equipment, professional coaching support, and specialized training materials suggests that the surveyed teachers perceived curriculum implementation as involving not only course-content design but also resource and professional-support requirements. In addition, many teachers indicated that enterprises should provide information on job-related physical fitness demands, and most of the eligible physical education teachers expressed willingness to participate in on-site investigations of information technology positions. These findings are broadly consistent with vocational-education perspectives emphasizing alignment between school-based training and occupational requirements and the potential contribution of enterprise participation to curriculum development and practice-based education [29,30]. Taken together, the teacher responses support further exploration of school–enterprise mechanisms through which job-demand information and relevant professional resources could inform occupation-related physical fitness education.

Third, the enterprise findings showed that some respondents perceived potential work-related consequences associated with physical fitness and identified several forms of support that enterprises could potentially provide to vocational colleges. In particular, respondents selected provision of job-related physical fitness standards, participation in curriculum design, and provision of physical fitness training scenarios during enterprise practice. Because the relevant questionnaire items assessed respondents’ perceptions and potential forms of support rather than verified organizational practices, these responses should not be interpreted as evidence of actual enterprise participation or established effects of physical fitness on work performance.

Although 15 of the 24 enterprise respondents reported paying at least some attention to candidates’ occupational physical fitness-related performance during recruitment, this descriptive finding should not be interpreted as support for using the proposed framework as an applicant-screening tool. The framework has not been validated for employment selection, fitness-for-employment determination, or prediction of individual job performance. Its potential application should instead be limited to inclusive occupational-health promotion, ergonomic support, curriculum development, and preparation for occupational demands. In particular, occupational physical fitness information should not be used to establish exclusionary hiring thresholds or to disadvantage individuals on the basis of disability, health status, or individual functional differences.

Fourth, the exploratory between-group analyses did not identify any occupational physical fitness dimension or individual indicator that remained statistically supported after false-discovery-rate correction across the 15 omnibus comparisons. Sustained concentration yielded the smallest raw p value, but this result did not survive multiplicity correction and was associated with a negligible effect size (ε² = 0.003). Accordingly, the nominal C2 finding should not be interpreted as practically meaningful or as robust evidence of stakeholder-specific priorities.

The full response distributions also showed substantial concentration toward the upper end of the five-point importance scale across most indicators and stakeholder groups. Although these ratings indicate that many of the proposed components were perceived as important, the limited dispersion suggests possible ceiling effects and reduced response discrimination. Consequently, small differences in medians or in the proportions selecting the highest response categories should not be interpreted as establishing a precise hierarchy of priorities. Possible acquiescence or generalized high-importance responding also cannot be excluded. These findings therefore support interpretation of the ratings as broad perceived importance rather than as evidence of statistical equivalence, stakeholder consensus, or a definitive ranking of intervention priorities.

It should be noted that the relationship between occupational sitting and mental health may be influenced by factors such as job type, task pressure, work autonomy, sleep quality, and individual physical activity levels.

Existing evidence regarding the association between occupational sitting and common mental-health symptoms remains inconclusive and may be influenced by job type, task pressure, work autonomy, sleep quality, and physical activity levels [31]. Accordingly, sedentary behavior is considered here as a contextual exposure characteristic of information technology learning and future work rather than as a demonstrated causal source of the psychological experiences reported by participants. The present study did not estimate an independent association between sedentary exposure and psychological outcomes; consequently, no confounder-adjusted effect estimate for such a relationship is reported.

These findings suggest that occupational physical fitness in information technology programs should not be simply understood as general sports ability. Unlike physically demanding occupations such as policing, firefighting, nursing, and manufacturing, the occupational context and stakeholder-rated priorities in information technology programs emphasize physical tolerance under prolonged sitting, regulatory capacity under sustained visual load, postural control during repetitive fine motor operations, and psychological adjustment under high cognitive pressure and task deadline pressure. Within the psychological-fitness domain, sustained concentration may also need to be considered in relation to the attentional fragmentation associated with digitally saturated learning and work environments. Protecting uninterrupted periods for cognitively demanding tasks and managing task-irrelevant digital interruptions may therefore represent relevant strategies for supporting sustained attention and cognitive functioning in such contexts [32]. Therefore, regular physical activity and the reduction of prolonged sedentary behavior should remain important foundations of physical fitness promotion for information technology students [33]. Building on these general health principles, the present findings further suggest that occupation-specific physical fitness and psychological fitness may warrant consideration in occupational physical fitness education for this population.

The integration of the questionnaire findings, interview themes, and expert content evaluation provided the empirical and contextual basis for proposing the three-dimensional framework of general physical fitness, occupation-specific physical fitness, and psychological fitness. The qualitative findings provided complementary contextual information: students described difficulties in addressing occupation-related physical discomfort through targeted exercise, teachers emphasized limitations in job-demand information and teaching resources, and enterprise personnel highlighted the relevance of sustained concentration, task-related stress, and physical tolerance in work contexts.

However, the proposed framework should be regarded as a preliminary conceptual organization of potentially relevant occupational physical fitness components rather than as a statistically validated latent structure. The internal-consistency and expert content-validity findings provide only preliminary measurement evidence and do not demonstrate that the proposed three-dimensional structure has been empirically established. Moreover, the high importance ratings across many indicators, limited response discrimination, and absence of multiplicity-adjusted between-group differences do not support a definitive hierarchy among the proposed components. Future studies should therefore examine the structural validity, stability, and applicability of the framework using larger and more balanced samples and, where appropriate, exploratory factor analysis, confirmatory factor analysis, structural equation modeling, objective physical-fitness assessments, and longitudinal or intervention designs.

The “general physical fitness–occupation-specific physical fitness–psychological fitness” framework proposed in this study has a certain degree of contextual explanatory value. General physical fitness provides the general physical foundation for students’ long-term learning and job adaptation. Occupation-specific physical fitness directly corresponds to characteristics of information technology programs, such as sedentary behavior, continuous screen exposure, sustained static postures, and fine motor operations. Psychological fitness reflects students’ ability to maintain stable performance during project-based tasks, internship pressure, and employment competition. It should be emphasized that this framework remains a preliminary conceptual framework rather than a mature scale model with verified structural validity. The internal-consistency coefficients and expert content-validity results provide preliminary measurement information for the proposed indicator set, but they do not establish the structural validity of the three-dimensional framework. Future studies should further examine this framework using exploratory factor analysis, confirmatory factor analysis, or structural equation modeling in larger samples and across more regions.

The integrated findings identified three complementary but conceptually distinct domains: student-reported experiences, stakeholder-rated priorities, and provision constraints. When considered together, these domains suggest incomplete alignment among student-reported experiences, stakeholder-rated priorities, and current educational and enterprise provision. Students reported physical and psychological experiences and limited exposure to occupation-specific training; teachers identified constraints related to job-demand information, specialized teaching resources, and curriculum implementation; and enterprise respondents identified potential forms of support but reported limited current involvement in curriculum-related activities. This pattern should not be interpreted as a formally demonstrated needs–provision gap because the study did not conduct a predefined needs assessment or gap analysis. Rather, it provides exploratory contextual evidence that closer coordination among student experiences, educational provision, and occupational input may warrant further investigation.

Based on these findings, physical education and health curricula in vocational colleges may consider shifting from “general exercise participation” toward “professional context adaptation.” In terms of curriculum content, occupational physical fitness modules may incorporate visual relaxation and protection, functional training for the neck, shoulder, and lower-back regions, core stability training, sedentary behavior interruption, postural management, sustained concentration training, and stress regulation training. In terms of teaching resources, specialized textbooks, training programs, and evaluation tools for information technology programs should be developed. In terms of collaborative mechanisms, enterprises may provide feedback on job task characteristics and health risks, which can then be jointly transformed by schools and professional physical fitness personnel into curriculum objectives and training content. These recommendations remain preliminary practical directions based on cross-sectional survey and interview data, and their actual effectiveness should be verified through future intervention studies.

## 5. Limitations

This study has several limitations.

First, this study used convenience sampling and snowball sampling, and the sample was mainly drawn from selected vocational colleges and related enterprises in Shanxi Province. Therefore, regional and institutional representativeness was limited, and the findings should not be generalized to all vocational colleges within Shanxi Province or to information technology students, teachers, and enterprise personnel across China. In addition, recruitment through class-, institution-, and enterprise-based channels may have resulted in correlated responses within recruitment units. Because cluster identifiers were not retained and clustering was not incorporated into the original statistical analyses, the statistical precision of some estimates and between-group comparisons may have been overstated. Future studies should prospectively record recruitment-unit identifiers and consider multilevel models, generalized estimating equations, or cluster-robust methods where appropriate.

Second, the sample sizes of the three groups were imbalanced. Students accounted for the majority of the sample, whereas the teacher and enterprise samples were relatively small. In particular, the enterprise sample included only 24 participants. Therefore, findings from the teacher and enterprise groups should be interpreted primarily as descriptive and exploratory evidence and should not be considered representative of the broader information technology industry or vocational education system. Moreover, nonsignificant between-group tests should not be interpreted as evidence of stakeholder equivalence or consensus, particularly given the small teacher and enterprise samples. In addition, the cross-stakeholder analysis involved multiple correlated outcomes. Although false-discovery-rate correction was applied across the 15 omnibus tests in the revised analysis, the small teacher and enterprise samples and the exploratory nature of these comparisons limit the precision and statistical power of the inferential results.

Third, this study adopted a cross-sectional design, and the data were mainly derived from self-reported questionnaires. The findings may therefore be affected by recall bias, social desirability bias, and common method bias, and causal relationships among perceived occupational physical fitness priorities, self-reported physical discomfort, self-reported psychological experiences, and curriculum provision cannot be determined. In addition, potential covariates relevant to any future analysis of sedentary exposure and psychological outcomes—such as validated sleep measures, detailed physical-activity characteristics, and other contextual factors—were not comprehensively measured; therefore, the present study cannot provide a confounder-adjusted estimate of such an association. Moreover, the physical-discomfort and psychological-experience items were study-specific, self-developed questions rather than validated symptom-specific or clinical instruments. Therefore, responses concerning dry eyes or blurred vision, cervical or lumbar soreness, sleep being affected, nervousness, difficulty concentrating, and related experiences should not be interpreted as clinical diagnoses or validated estimates of digital eye strain, musculoskeletal disorders, anxiety, insomnia, sleep disturbance, or other mental health conditions. Some self-reported symptom and experience items also did not specify a fixed retrospective recall period or numerical thresholds for qualitative frequency categories such as “frequently” and “occasionally.” Accordingly, the resulting percentages should be interpreted as descriptive response proportions rather than period-prevalence estimates. Weekly exercise was assessed using broad duration categories only and did not capture exercise intensity, frequency, moderate-to-vigorous physical activity, or sedentary time. Therefore, the study cannot determine whether participants met established physical-activity recommendations.

Future studies should use prespecified recall windows and validated symptom-specific instruments and, where appropriate, incorporate objective or clinical assessments of symptoms and standardized measures of physical activity intensity, frequency, and duration to improve measurement validity, temporal interpretability, and comparability. Future studies may also complement self-reported discomfort with objective technologies, including wearable physiological sensors and inertial measurement units, to characterize postural exposure and movement patterns during prolonged computer-based tasks. Such sensor-based assessments should use standardized and validated measurement protocols to improve the reliability and comparability of the resulting data [34]. With prospectively collected longitudinal and multimodal data, future studies could further explore machine-learning and artificial-intelligence approaches to estimate individual risk of posture-related discomfort and to inform personalized preventive strategies [35,36]. Such predictive models would require adequate sample sizes, clearly defined outcomes, appropriate internal and external validation, and careful attention to model interpretability and data privacy before practical application.

Fourth, the preliminary conceptual framework proposed in this study was constructed exploratorily based primarily on literature analysis, expert content validity evaluation, questionnaire survey data, and interview data. It has not yet been validated through exploratory factor analysis, confirmatory factor analysis, structural equation modeling, or objective physical fitness testing. In addition, the content-validity evaluation was based on a panel of only eight experts; consequently, the I-CVI and S-CVI/Ave values are panel-dependent estimates that may be sensitive to individual expert ratings and should be regarded as preliminary evidence of expert agreement rather than definitive validation of the indicator framework. Predictive validity was not evaluated because the present study did not collect a prospective external criterion or objective occupational-performance outcome suitable for predictive modeling. In addition, although the 12 core importance-rating items used for cross-stakeholder comparisons had identical item wording, response options, coding, and dimensional composition across the three questionnaire versions, the introductory stems were contextualized for each stakeholder group, and formal measurement invariance was not examined. Consequently, cross-stakeholder score comparisons should be interpreted as exploratory rather than as evidence of full cross-group measurement equivalence. The importance ratings also showed substantial concentration toward the upper end of the five-point response scale across most indicators, suggesting possible ceiling effects and limited response discrimination. Because the items assessed individually presented and generally desirable physical and psychological attributes, acquiescence or generalized high-importance responding cannot be excluded. Accordingly, small differences in medians or in the proportions selecting high response categories should not be interpreted as establishing a definitive hierarchy of occupational physical fitness priorities. Future instrument-development studies should further evaluate item discrimination and consider measurement approaches that can better differentiate relative priorities among components that are all perceived as important. Therefore, the framework should currently be understood as a preliminary conceptual model of the components of occupational physical fitness qualities for information technology programs, and further examination is needed in larger-scale studies with more balanced samples, as well as in longitudinal or intervention studies.

Fifth, this study did not conduct a formal needs assessment or predefined gap analysis. Importance ratings, self-reported symptoms or experiences, training participation, and provision-related constraints represent distinct constructs and should not be interpreted as interchangeable evidence of an unmet occupational physical fitness need. Accordingly, the identified priorities and proposed curriculum directions should be regarded as exploratory and require confirmation through dedicated needs-assessment and intervention studies.

Sixth, although interview participants were purposively selected across the three stakeholder groups to capture variation in relevant educational and occupational backgrounds and experiences, detailed individual demographic characteristics of the qualitative sample were not systematically recorded. Therefore, the extent of demographic variation within the interview sample cannot be independently evaluated, which limits assessment of the transferability of the qualitative findings.

Seventh, the original interview audio files, verbatim transcripts, field notes, original coding records, and detailed interview-procedure documentation were no longer available at the time of manuscript revision. The qualitative findings therefore rely on the thematic results preserved in the contemporaneous study-report documentation, and several COREQ-relevant procedural details could not be independently verified or retrospectively reconstructed. This also prevented independent re-analysis of the original qualitative material and limits the auditability, confirmability, and methodological depth of the qualitative component.

Finally, although the total number of returned questionnaires and the final analytical sample were available, the historical pre-cleaning disposition records were no longer available at the time of revision. Therefore, the stakeholder-specific numbers of returned questionnaires and the numbers excluded under each individual screening criterion could not be retrospectively reconstructed. Platform-level counts of individuals who accessed the survey, provided consent, or began but did not complete the questionnaire were also unavailable. This limits a detailed quantitative assessment of potential nonresponse and data-cleaning-related selection bias.

## 6. Conclusions

Based on questionnaire survey and interview data from information technology students, teachers, and enterprise personnel recruited in Shanxi Province, China, this study found that students reported several forms of physical discomfort, while weekly exercise duration was most commonly reported as 1–3 h/week. The survey and interview findings also suggested limited incorporation of occupation-specific physical fitness training into the educational contexts examined. The teacher and enterprise findings further indicated perceived constraints related to access to job-demand information, the availability of occupation-specific training resources, and school–enterprise collaboration. Based on literature, expert evaluation, questionnaire data, and interview findings, this study exploratorily proposed a preliminary conceptual framework of occupational physical fitness for information technology programs, consisting of general physical fitness, occupation-specific physical fitness, and psychological fitness. This framework may provide a preliminary reference for physical education and health curriculum development in vocational-college contexts similar to those examined in this study. However, because this study used convenience sampling, included relatively small teacher and enterprise samples, and lacked structural validity testing and objective measurement verification, the proposed framework and related conclusions require further examination in larger, more diverse samples and through longitudinal or intervention research. The present findings reflect stakeholder-perceived priorities and related experiential and provision contexts rather than formally established unmet needs, as no formal needs assessment or gap analysis was conducted.

## 7. Practical implications

Based on the findings of this study, occupational physical fitness training for information technology programs may be explored from three aspects: curriculum content, teaching resources, and school–enterprise collaboration. It should be emphasized that these recommendations are mainly derived from cross-sectional survey and interview data and have not yet been verified through intervention studies. Therefore, they should be regarded as preliminary directions for curriculum reform rather than as intervention programs with established effectiveness. These practical implications are intended exclusively to support inclusive education, occupational-health promotion, ergonomic support, and student well-being. The proposed framework is neither intended nor validated for applicant screening, hiring decisions, exclusion from employment, or fitness-for-employment certification.

First, vocational colleges in contexts similar to those examined in this study may consider establishing occupational physical fitness modules within physical education and health curricula, incorporating commonly reported concerns among information technology students, such as eye-related discomfort, neck or lumbar soreness, sedentary fatigue, and psychological experiences associated with intensive study and project demands, into course content. The curriculum may include eye relaxation training, functional training for the neck, shoulder, and lower-back regions, core stability training, sedentary behavior interruption training, postural management training, and stress regulation training. For sedentary exposure, strategies such as active breaks, sit–stand transitions, and regular activity interruptions from workplace sedentary behavior reduction interventions may be referenced [37]. For visual protection, principles from digital eye strain management, such as regular screen breaks, optimization of the visual environment, and adjustment of eye-use behaviors, may be adopted [1,12,14]. For neck, shoulder, and lower-back discomfort, curriculum design may be informed by ergonomics and exercise intervention programs [12,14,38,39].

Second, schools should strengthen the development of teaching resources for occupational physical fitness, including developing specialized teaching materials, providing basic training equipment, establishing occupational physical fitness training areas, and offering relevant training for teachers. For teachers who lack job-related experience, understanding of the physical fitness demands of information technology positions may be improved through enterprise investigations, job experience activities, and collaborative lesson preparation.

Finally, school–enterprise collaboration may be used to support inclusive occupational-health education and preparation for information technology work contexts [29,30]. Enterprises may provide schools with information on job-task characteristics, ergonomic exposures, and health-support needs, and may contribute to curriculum design, internship guidance, and workplace-health education. Such collaboration should focus on improving learning environments, ergonomic awareness, health-support resources, and students’ capacity to manage occupational demands rather than on evaluating eligibility for employment. Any educational or health-related assessment should be voluntary, supportive, and non-exclusionary and should not be used as a hiring criterion or fitness-for-employment test.

## Supporting information

S1 Questionnaire Questionnaire (English Version). (DOCX)

S1 Data De-identified final analytical dataset. (XLSX)

S2 Data Expert Content Validity Evaluation. (XLSX)

S1 Checklist Consolidated criteria for reporting qualitative research (COREQ) checklist for the semi-structured interview component. (DOCX)

S2 Checklist STROBE Checklist for the quantitative cross-sectional component. (DOCX)

S3 Checklist Inclusivity in global research questionnaire. (DOCX)

S1 Table Cross-version mapping of the common occupational physical fitness importance-rating items. (DOCX)

S2 Table Data dictionary and coding specification for the final analytical dataset. (XLSX)

S3 Table Operational definitions, reference/recall periods, response categories, and reporting rules for principal questionnaire outcomes. (DOCX)

S4 Table Mixed-methods joint display linking quantitative findings, qualitative themes, and integrated interpretations. (DOCX)

S5 Table Full response distributions for the 12 occupational physical fitness importance-rating indicators by stakeholder group. (DOCX)

S1 File Reproducible statistical analysis syntax. (TXT)

S2 File Data processing, de-identification, and reproducibility README. (TXT)
