# Supplementary material for: Perceived occupational physical fitness priorities and a preliminary conceptual framework for information technology students at selected vocational colleges in Shanxi Province, China: An exploratory cross-sectional mixed-methods study
Source: PLoS One. 2026 Nov 10;21(11):e0362309. doi: 10.1371/journal.pone.0362309 (PMC13750592; doi:10.1371/journal.pone.0362309)
Supplement: Supplementary file 1 [file pone.0362309.s001.docx]

# Questionnaire on Occupational Physical Fitness Qualities and Training Needs of Information Technology Students

# （University/College Teacher Version QA）

Dear Teacher,

Hello!

To gain an in-depth understanding of the occupational physical fitness quality structure and current training status of highly skilled information technology talents in the new era, and to explore training pathways for information technology talents that meet the needs of occupational environments and high-quality development, we are conducting this survey. Your valuable opinions are of great significance for improving the training system for information technology talents and promoting industry development. This questionnaire is anonymous, and the data will be used only for academic research. Please answer the questions according to your actual situation.

Thank you for taking the time to participate in this survey!

Note: Occupational physical fitness qualities refer to the comprehensive qualities that workers possess in terms of physical function, motor ability, and physiological adaptability in order to meet the work demands of specific occupational positions. They constitute the core physical foundation for ensuring efficient occupational performance, reducing the risk of occupational injury, and maintaining long-term occupational competence.

QA1. What is your gender?

A. Male

B. Female

QA2. What is your age?

A. 20–30 years

B. 31–40 years

C. 41–50 years

D. Over 50 years

QA3. What type of institution do you work at?

A. General undergraduate institution

B. Vocational undergraduate institution

C. Higher vocational college / junior college

D. Other, please specify: ______

QA4. How many years of teaching experience do you have?

A. 1–3 years

B. 4–6 years

C. 7–10 years

D. More than 10 years

QA5. What is your professional title?

A. Teaching assistant

B. Lecturer

C. Associate professor

D. Professor

QA6. What is your main teaching field?

A. Basic professional courses, such as Computer Organization Principles and Data Structures

B. Core professional courses, such as Practical Software Development and Network Attack and Defense

C. General education courses, such as Advanced Mathematics and College English

D. Physical education courses, including courses related to physical fitness training

E. Practical training courses, such as enterprise project training and equipment operation training

F. Other, please specify: ______

If you selected D, physical education courses / physical fitness-related courses, please answer QA7a–QA9a.

QA7a. When designing physical fitness courses for information technology students, what are the greatest difficulties you encounter? Multiple responses are allowed.

A. Lack of specific data on the physical fitness demands of information technology positions, such as sedentary duration and movement intensity

B. Existing physical fitness training methods are difficult to match professional job scenarios

C. Students show low interest in courses integrating “professional learning + physical fitness”

D. The school does not provide targeted training equipment, such as lower back support training devices or eye relaxation equipment

E. Other, please specify: ______

QA8a. In your courses, have you ever tried to design physical fitness training content based on the job demands of information technology positions, such as lower back training for prolonged sitting or eye relaxation training for prolonged screen viewing?

A. Frequently, such as more than three times per semester

B. Occasionally, such as one to two times per semester

C. Never

D. I would like to, but I lack references on job-related physical fitness demands

QA9a. If given the opportunity, would you be willing to participate in on-site investigations of enterprise information technology positions to understand their actual physical fitness demands and optimize course design?

A. Very willing

B. Willing

C. Neutral

D. Unwilling

E. Very unwilling, please explain: ______

If you did not select D, that is, if you are not a physical fitness-related course teacher, please answer QA7b–QA9b.

QA7b. Do you have work experience in enterprise information technology-related positions?

A. Yes, years of work experience: ______ years

B. No

QA8b. During teaching, have you observed that students’ physical fitness-related problems, such as sedentary fatigue or visual discomfort, affect their professional learning or practical efficiency?

A. Frequently observed

B. Occasionally observed

C. Rarely observed

D. Never observed

QA9b. Do you think the definition of “occupational physical fitness” provided at the beginning of this questionnaire covers the core needs of information technology positions?

A. Fully covers them

B. Basically covers them

C. Partially covers them

D. Does not cover them, please add: ______

E. Not sure

QA10. Does your institution offer specialized occupational physical fitness training courses for information technology students?

A. Yes, course frequency: ______

B. No

C. Planned

QA11. How would you evaluate the overall level of occupational physical fitness qualities among information technology students in current vocational colleges?

A. Excellent

B. Good

C. Average

D. Poor

E. Not sure

QA12. In daily teaching, what physical fitness-related problems do you most often observe among students during information technology-related practical operations, such as long-term computer operation and equipment debugging? Multiple responses are allowed.

A. Cervical or lumbar discomfort

B. Decline in vision

C. Strain of hand joints

D. Difficulty concentrating

E. Insufficient endurance

F. Other, please specify: ______

QA13. What core occupational physical fitness qualities do you think highly skilled information technology talents should possess? Multiple responses are allowed.

A. Sedentary tolerance

The comprehensive ability to maintain stable physical function and resist local muscle fatigue and physiological discomfort through implicit muscular support, blood circulation regulation, and physiological tolerance while maintaining a seated posture for a prolonged period, usually ≥4 hours.

B. Fine motor ability of the hands

C. Visual fatigue tolerance

D. Body posture regulation ability

E. Emergency response speed

F. Other, please specify: ______

QA14. Based on the three-level structural model of “general physical fitness–occupation-specific physical fitness–psychological fitness,” please evaluate the importance of the following occupational physical fitness elements for the future career development of information technology students.

Scoring instructions: Please select one score for each item:

1 = Not important at all; 2 = Not important; 3 = Moderately important; 4 = Important; 5 = Very important.

| **Dimension** | **Specific Element** | **Description** | **Importance Rating** |
| --- | --- | --- | --- |
| General physical fitness (A) | A1 Cardiorespiratory endurance | Basic physical fitness reserve for maintaining long working hours; affects overall work sustainability and attendance stability. | □ 1 □ 2 □ 3 □ 4 □ 5 |
|  | A2 Muscular strength | Strength of the lower back and core muscles needed to maintain body stability; helps prevent lumbar and cervical disorders and reduce sedentary strain. | □ 1 □ 2 □ 3 □ 4 □ 5 |
|  | A3 Flexibility | Range of joint motion and muscle extensibility; facilitates relaxation during work breaks and reduces the risk of muscle strain. | □ 1 □ 2 □ 3 □ 4 □ 5 |
|  | A4 Bodily coordination | Movement fluency and balance control ability; affects equipment operation efficiency and the precision of hand–eye coordination. | □ 1 □ 2 □ 3 □ 4 □ 5 |
| Occupation-specific physical fitness (B) | B1 Sedentary tolerance | Muscular endurance and physiological adaptability when maintaining a seated posture for a long period, ≥4 hours; directly affects daily office efficiency and comfort. | □ 1 □ 2 □ 3 □ 4 □ 5 |
|  | B2 Visual load tolerance | Ability to maintain visual function after prolonged screen viewing, such as programming, design, and debugging; related to work accuracy and eye health. | □ 1 □ 2 □ 3 □ 4 □ 5 |
|  | B3 Fine motor operation endurance | Endurance of the fingers and wrists during prolonged precise operations, such as keyboard input and equipment debugging; affects code quality and operational accuracy. | □ 1 □ 2 □ 3 □ 4 □ 5 |
|  | B4 Static postural endurance | Stability of the cervical and lumbar regions while maintaining a working posture; influences occupational health status and related medical costs. | □ 1 □ 2 □ 3 □ 4 □ 5 |
| Psychological fitness (C) | C1 Stress resistance | Psychological resilience in coping with high-intensity work pressure and pressure during critical project periods; affects employee stability and work output. | □ 1 □ 2 □ 3 □ 4 □ 5 |
|  | C2 Sustained concentration | Ability to maintain attention for a long period, such as more than two consecutive hours of programming; directly determines the quality of complex task completion. | □ 1 □ 2 □ 3 □ 4 □ 5 |
|  | C3 Emotional regulation | Ability to manage negative emotions such as anxiety, frustration, and irritability at work; affects teamwork and turnover rate. | □ 1 □ 2 □ 3 □ 4 □ 5 |
|  | C4 Emergency response capacity | Ability to make rapid decisions and execute actions when facing sudden technical problems, such as system failures or cyberattacks; related to handling efficiency and customer satisfaction. | □ 1 □ 2 □ 3 □ 4 □ 5 |

QA15. What do you think are the main factors currently affecting the improvement of occupational physical fitness qualities among information technology students? Multiple responses are allowed.

A. Students do not attach sufficient importance to it

B. Schools lack professional training resources

C. Occupational physical fitness-related content is absent from the curriculum system

D. Teachers lack the ability to teach occupational physical fitness

E. Other, please specify: ______

QA16. How necessary do you think it is to develop students’ occupational physical fitness?

A. Completely unnecessary

B. Unnecessary

C. Uncertain

D. Necessary

E. Absolutely necessary

QA17. Do you think it is necessary to clearly define occupational physical fitness training objectives in the talent training program for information technology majors?

A. Very necessary

B. Necessary

C. Neutral

D. Unnecessary

E. Completely unnecessary

QA18. If the school plans to provide occupational physical fitness training for information technology students, what forms of training would you prefer? Multiple responses are allowed.

A. Specialized physical fitness training courses

B. Physical fitness training integrated into professional practical teaching

C. Extracurricular physical fitness development activities

D. Online physical fitness guidance courses

E. Other, please specify: ______

QA19. If the school plans to provide occupational physical fitness training for information technology students, what content would you prefer it to focus on? Multiple responses are allowed.

A. General physical fitness, such as strength, speed, endurance, flexibility, and agility

B. Physical fitness combined with professional characteristics, that is, targeted training and development of specific physical fitness based on future job demands

C. Health education, such as basic health knowledge, occupational disease prevention, injury prevention, and injury management

D. Not sure

QA20. What teaching resources do you think are most needed in occupational physical fitness teaching? Multiple responses are allowed.

A. Specialized occupational physical fitness training materials

B. Physical fitness training equipment

C. Professional physical fitness coaches

D. Case database for occupational physical fitness teaching

E. Other, please specify: ______

QA21. Are you willing to participate in teaching training related to occupational physical fitness to improve your own occupational physical fitness teaching ability?

A. Very willing

B. Willing

C. Neutral

D. Unwilling

E. Very unwilling

QA22. Through which channels do you mainly learn about occupational physical fitness?

A. Policy documents

B. The Internet

C. Professional training

D. Other channels

QA23. What role do you think enterprises should play in cultivating occupational physical fitness qualities among information technology students? Multiple responses are allowed.

A. Provide standards for job-related physical fitness demands

B. Participate in occupational physical fitness curriculum design

C. Provide physical fitness training scenarios in enterprise practice

D. No participation is needed

E. Other, please specify: ______

QA24. Do you have any other valuable suggestions regarding the occupational physical fitness quality structure and training pathways for highly skilled information technology talents in vocational colleges?

___________________________________________________	___

Thank you again for your support and cooperation in this survey!

# Questionnaire on Occupational Physical Fitness Qualities and Training Needs of Information Technology Students

# （University/College Student Version QB）

Dear Student,

Hello!

To gain an in-depth understanding of the occupational physical fitness qualities of highly skilled information technology talents in the new era and to explore a training model that better supports the development of higher vocational information technology students, we are conducting this survey. Your honest feedback is very important for optimizing curriculum design, improving practical teaching, and enhancing the quality of talent training. This questionnaire is anonymous, and the data will be used only for academic research. Please feel free to answer the questions according to your actual situation.

Thank you for taking the time to participate!

Note: Occupational physical fitness qualities refer to the comprehensive qualities that workers possess in terms of physical function, motor ability, and physiological adaptability in order to meet the work demands of specific occupational positions. They constitute the core physical foundation for ensuring efficient occupational performance, reducing the risk of occupational injury, and maintaining long-term occupational competence.

QB1. What is your gender?

A. Male

B. Female

QB2. What is your current year of study?

A. First year

B. Second year

C. Third year

D. Fourth year / internship stage

E. Graduated

QB3. What is your specific field within information technology?

A. Software development and engineering

B. Communication-related fields, such as communication software technology and 5G communication software technology

C. Network technology, such as intelligent Internet technology, network construction, and cybersecurity

D. Cloud computing technology and applications

E. Blockchain technology

F. Big data and artificial intelligence

G. Internet of Things technology

H. Digital media technology, such as UI/UX design and post-production for film and video

I. Information technology operation and maintenance, such as equipment maintenance and system administration

J. Other, please specify: ______

QB4. Approximately how many hours per week do you spend on professional practical operations, such as programming, equipment debugging, and design?

A. Less than 10 hours

B. 10–20 hours

C. 20–30 hours

D. More than 30 hours

QB5. Have you had any internship experience in an information technology-related industry?

A. Yes, internship duration: ______ months

B. No

QB6. On average, how much time do you spend on physical exercise each week, such as running, fitness training, or ball games?

A. Almost no exercise

B. 1–3 hours

C. 3–5 hours

D. 5–7 hours

E. More than 7 hours

QB7. What types of sports or exercise do you usually enjoy? Multiple responses are allowed.

A. Running / brisk walking

B. Ball games, such as basketball and football

C. Fitness / strength training

D. Yoga / Pilates / stretching

E. Swimming

F. Cycling / outdoor sports

G. Martial arts / traditional ethnic sports

H. I do not like exercise

I. Other, please specify: ______

QB8. After professional practical operations, such as long-term computer use or equipment debugging, do you experience physical discomfort?

A. Frequently

B. Occasionally

C. Rarely

D. Never

QB9. If you experience physical discomfort, what are the main symptoms? Multiple responses are allowed. If you do not experience discomfort, please select “None.”

A. Cervical or lumbar soreness

B. Dry eyes or blurred vision

C. Soreness or swelling of the wrist or finger joints

D. Dizziness or difficulty concentrating

E. Physical fatigue or insufficient endurance

F. None

G. Other, please specify: ______

QB10. During professional learning or practice, have you experienced any of the following psychological states? Multiple responses are allowed.

Friendly reminder: These are normal experiences. Your honest feedback will help improve teaching.

A. Feeling so anxious before exams or project deadlines that your sleep is affected

B. Feeling mentally “shut down” and completely unable to concentrate after studying or programming continuously for 2 hours

C. Becoming easily irritated or wanting to give up when encountering bugs or technical problems

D. Overeating or losing appetite when under heavy learning pressure

E. Feeling tired of professional learning and losing interest

F. Frequently feeling nervous because of concerns about employment or internship performance

G. Losing emotional control in team assignments because of disagreements

H. The above situations rarely occur

I. Other, please specify: ______

QB11. When you experience the above psychological stress or negative emotions, what methods do you usually use to relieve them? Multiple responses are allowed.

A. Exercise / physical activity

B. Talking with friends or family members

C. Listening to music / watching videos to relax

D. Continuing to push through without taking any action

E. Overeating or using other unhealthy methods

F. Self-regulation through methods such as deep breathing and meditation

G. Seeking help from psychological counseling services or a counselor

H. Other, please specify: ______

QB12. Do you think the occupational physical fitness qualities required by information technology students are important for future work?

A. Very important

B. Relatively important

C. Average

D. Not very important

E. Not important at all

QB13. Do you understand what occupational diseases may exist in the work you may do in the future?

A. Understand very well

B. Generally understand

C. Do not understand very well

D. Do not understand at all

QB14. What physical qualities do you think will be required for your future job? Multiple responses are allowed.

A. Strength

B. Speed

C. Endurance

D. Coordination

E. Other, please specify: ______

QB15. The table below lists three types of occupational physical fitness qualities required for information technology positions. Based on your learning experience and understanding of future work, please evaluate the importance of each quality.

Scoring instructions: Please select one score for each item:

1 = Not important at all; 2 = Not important; 3 = Moderately important; 4 = Important; 5 = Very important.

| **Dimension** | **Specific Element** | **Description** | **Importance Rating** |
| --- | --- | --- | --- |
| General physical fitness (A) | A1 Cardiorespiratory endurance | Basic physical fitness reserve for maintaining long working hours; affects overall work sustainability and attendance stability. | □ 1 □ 2 □ 3 □ 4 □ 5 |
|  | A2 Muscular strength | Strength of the lower back and core muscles needed to maintain body stability; helps prevent lumbar and cervical disorders and reduce sedentary strain. | □ 1 □ 2 □ 3 □ 4 □ 5 |
|  | A3 Flexibility | Range of joint motion and muscle extensibility; facilitates relaxation during work breaks and reduces the risk of muscle strain. | □ 1 □ 2 □ 3 □ 4 □ 5 |
|  | A4 Bodily coordination | Movement fluency and balance control ability; affects equipment operation efficiency and the precision of hand–eye coordination. | □ 1 □ 2 □ 3 □ 4 □ 5 |
| Occupation-specific physical fitness (B) | B1 Sedentary tolerance | Muscular endurance and physiological adaptability when maintaining a seated posture for a long period, ≥4 hours; directly affects daily office efficiency and comfort. | □ 1 □ 2 □ 3 □ 4 □ 5 |
|  | B2 Visual load tolerance | Ability to maintain visual function after prolonged screen viewing, such as programming, design, and debugging; related to work accuracy and eye health. | □ 1 □ 2 □ 3 □ 4 □ 5 |
|  | B3 Fine motor operation endurance | Endurance of the fingers and wrists during prolonged precise operations, such as keyboard input and equipment debugging; affects code quality and operational accuracy. | □ 1 □ 2 □ 3 □ 4 □ 5 |
|  | B4 Static postural endurance | Stability of the cervical and lumbar regions while maintaining a working posture; influences occupational health status and related medical costs. | □ 1 □ 2 □ 3 □ 4 □ 5 |
| Psychological fitness (C) | C1 Stress resistance | Psychological resilience in coping with high-intensity work pressure and pressure during critical project periods; affects employee stability and work output. | □ 1 □ 2 □ 3 □ 4 □ 5 |
|  | C2 Sustained concentration | Ability to maintain attention for a long period, such as more than two consecutive hours of programming; directly determines the quality of complex task completion. | □ 1 □ 2 □ 3 □ 4 □ 5 |
|  | C3 Emotional regulation | Ability to manage negative emotions such as anxiety, frustration, and irritability at work; affects teamwork and turnover rate. | □ 1 □ 2 □ 3 □ 4 □ 5 |
|  | C4 Emergency response capacity | Ability to make rapid decisions and execute actions when facing sudden technical problems, such as system failures or cyberattacks; related to handling efficiency and customer satisfaction. | □ 1 □ 2 □ 3 □ 4 □ 5 |

QB16. During periods of intensive study or project work, what is your usual psychological state? Multiple responses are allowed.

A. I can stay focused, and the pressure is manageable

B. I easily become anxious, and my sleep is affected

C. It is difficult for me to maintain attention for more than 2 hours

D. I relieve stress through exercise or other methods

E. Other, please specify: ______

QB17. How satisfied are you with your current occupational physical fitness qualities, such as sedentary tolerance and hand coordination?

A. Very satisfied

B. Relatively satisfied

C. Average

D. Not very satisfied

E. Very dissatisfied

QB18. What do you think are the main reasons affecting the improvement of your occupational physical fitness qualities? Multiple responses are allowed.

A. Lack of time, due to heavy academic work or internships

B. Not knowing how to train scientifically

C. Lack of suitable training venues or resources

D. Not attaching enough importance to it

E. Other, please specify: ______

QB19. To what extent do you think good occupational physical fitness helps with professional skill learning?

A. Helps a lot, such as improving programming efficiency when physical fitness is good

B. Helps to some extent

C. Has little relationship

D. Does not help

E. Not sure

QB20. Can your school’s current occupational physical fitness teaching resources meet your needs for occupational physical fitness development?

A. Can fully meet the needs

B. Can generally meet the needs

C. Average

D. Cannot meet the needs very well

E. Cannot meet the needs

QB21. Would you like your school to offer occupational physical fitness-related courses for information technology students?

A. Strongly hope so

B. Hope so

C. Neutral

D. Do not really hope so

E. Strongly do not hope so

QB22. If occupational physical fitness courses are offered, which course formats would you prefer? Multiple responses are allowed.

A. Offline practical training, such as targeted physical fitness exercises

B. Online guidance courses, such as home training videos

C. Physical fitness training integrated into professional practice, such as relaxation training during operation breaks

D. Lectures on physical fitness knowledge, such as occupational health protection knowledge

E. Other, please specify: ______

QB23. Which abilities would you most like to improve through occupational physical fitness courses? Multiple responses are allowed.

A. Ability to relieve lower back discomfort after prolonged sitting

B. Techniques for relieving eye fatigue

C. Fine motor endurance of the hands

D. Methods for correcting body posture

E. Emergency response speed

F. Other, please specify: ______

QB24. Do you have any other suggestions regarding the cultivation of occupational physical fitness qualities among highly skilled information technology talents in vocational colleges?

Thank you again for your support and cooperation in this survey!

# Questionnaire on Occupational Physical Fitness Qualities and Training Needs of Information Technology Students

# （Enterprise Version QC）

Dear Respondent,

Hello!

To gain an in-depth understanding of the occupational physical fitness qualities of highly skilled information technology talents in the new era and to improve the talent training system for information technology programs, we are conducting this survey. Your responses are of great significance to this research. This questionnaire is anonymous, and the data will be used only for academic research. Please answer the questions according to your actual situation.

Thank you for your support and cooperation!

Note: Occupational physical fitness qualities refer to the comprehensive qualities that workers possess in terms of physical function, motor ability, and physiological adaptability in order to meet the work demands of specific occupational positions. They constitute the core physical foundation for ensuring efficient occupational performance, reducing the risk of occupational injury, and maintaining long-term occupational competence.

QC1. What is the specific industry field of your enterprise?

A. Software development and services, such as app development and outsourced software development

B. Network and information security, such as network protection and data security

C. Internet of Things and smart devices, such as IoT system development and smart hardware operation and maintenance

D. Artificial intelligence and big data, such as AI algorithm development and big data analysis

E. Digital media and design, such as film and video production and game development

F. Information technology operation and maintenance services, such as IT system maintenance and equipment inspection/repair

G. Other, please specify: ______

QC2. What is the size of your enterprise, based on the number of employees?

A. 50 employees or fewer

B. 51–200 employees

C. 201–500 employees

D. More than 501 employees

QC3. Approximately how many graduates from vocational college information technology programs has your enterprise recruited in the past three years?

A. 5 or fewer

B. 6–15

C. 16–30

D. 31 or more

QC4. What is your main area of responsibility in the enterprise?

A. Human resources recruitment and training

B. Technical department management

C. Front-line technical research and development / operation and maintenance

D. Other, please specify: ______

QC5. In your opinion, can the occupational physical fitness qualities of current vocational college information technology graduates meet job demands after employment?

A. Fully meet the demands

B. Basically meet the demands

C. Average

D. Do not fully meet the demands

E. Do not meet the demands at all

QC6. In daily work, how often have you observed that information technology employees, including graduates, are affected at work by physical fitness-related problems?

A. Frequently

B. Occasionally

C. Rarely

D. Never

QC7. What occupational physical fitness qualities do you think are most needed for information technology positions? Multiple responses are allowed.

A. Sedentary tolerance, that is, adapting to long periods of office work

B. Fine motor ability of the hands, such as keyboard operation and equipment debugging

C. Visual fatigue tolerance, that is, adapting to prolonged screen viewing

D. Body posture regulation ability, that is, avoiding sedentary strain

E. Emergency response speed, that is, responding to sudden technical problems

F. Other, please specify: ______

QC8. Based on the actual work demands of information technology positions in your enterprise, please evaluate the importance of the following three types of occupational physical fitness qualities and their specific elements for employees’ work performance.

Scoring instructions: Please select one score for each item:

1 = Not important at all; 2 = Not important; 3 = Moderately important; 4 = Important; 5 = Very important.

| **Dimension** | **Specific Element** | **Description** | **Importance Rating** |
| --- | --- | --- | --- |
| General physical fitness (A) | A1 Cardiorespiratory endurance | Basic physical fitness reserve for maintaining long working hours; affects overall work sustainability and attendance stability. | □ 1 □ 2 □ 3 □ 4 □ 5 |
|  | A2 Muscular strength | Strength of the lower back and core muscles needed to maintain body stability; helps prevent lumbar and cervical disorders and reduce sedentary strain. | □ 1 □ 2 □ 3 □ 4 □ 5 |
|  | A3 Flexibility | Range of joint motion and muscle extensibility; facilitates relaxation during work breaks and reduces the risk of muscle strain. | □ 1 □ 2 □ 3 □ 4 □ 5 |
|  | A4 Bodily coordination | Movement fluency and balance control ability; affects equipment operation efficiency and the precision of hand–eye coordination. | □ 1 □ 2 □ 3 □ 4 □ 5 |
| Occupation-specific physical fitness (B) | B1 Sedentary tolerance | Muscular endurance and physiological adaptability when maintaining a seated posture for a long period, ≥4 hours; directly affects daily office efficiency and comfort. | □ 1 □ 2 □ 3 □ 4 □ 5 |
|  | B2 Visual load tolerance | Ability to maintain visual function after prolonged screen viewing, such as programming, design, and debugging; related to work accuracy and eye health. | □ 1 □ 2 □ 3 □ 4 □ 5 |
|  | B3 Fine motor operation endurance | Endurance of the fingers and wrists during prolonged precise operations, such as keyboard input and equipment debugging; affects code quality and operational accuracy. | □ 1 □ 2 □ 3 □ 4 □ 5 |
|  | B4 Static postural endurance | Stability of the cervical and lumbar regions while maintaining a working posture; influences occupational health status and related medical costs. | □ 1 □ 2 □ 3 □ 4 □ 5 |
| Psychological fitness (C) | C1 Stress resistance | Psychological resilience in coping with high-intensity work pressure and pressure during critical project periods; affects employee stability and work output. | □ 1 □ 2 □ 3 □ 4 □ 5 |
|  | C2 Sustained concentration | Ability to maintain attention for a long period, such as more than two consecutive hours of programming; directly determines the quality of complex task completion. | □ 1 □ 2 □ 3 □ 4 □ 5 |
|  | C3 Emotional regulation | Ability to manage negative emotions such as anxiety, frustration, and irritability at work; affects teamwork and turnover rate. | □ 1 □ 2 □ 3 □ 4 □ 5 |
|  | C4 Emergency response capacity | Ability to make rapid decisions and execute actions when facing sudden technical problems, such as system failures or cyberattacks; related to handling efficiency and customer satisfaction. | □ 1 □ 2 □ 3 □ 4 □ 5 |

QC9. What are the common problems among information technology employees in your enterprise caused by insufficient physical fitness? Multiple responses are allowed.

A. Decreased work efficiency

B. Increased frequency of taking leave, such as due to cervical or lumbar problems

C. Increased rate of operational errors

D. No obvious problems

E. Other, please specify: ______

QC10. When recruiting information technology talents, does your enterprise pay attention to their occupational physical fitness-related performance?

A. Pays very close attention

B. Pays some attention

C. Average

D. Pays little attention

E. Pays no attention at all

QC11. To what extent do you think the occupational physical fitness needs differ across different information technology positions, such as development, operation and maintenance, and design?

A. Very large differences

B. Some differences

C. Small differences

D. No differences

E. Not sure

QC12. Do you think it is necessary for vocational colleges to provide specialized occupational physical fitness training when cultivating information technology students?

A. Very necessary

B. Necessary

C. Neutral

D. Unnecessary

E. Completely unnecessary

QC13. If vocational colleges invite enterprises to participate in occupational physical fitness training for information technology students, what kinds of support do you think enterprises could provide? Multiple responses are allowed.

A. Provide standards for job-related physical fitness demands

B. Participate in the design of physical fitness training courses

C. Provide physical fitness training scenarios in enterprise practice, such as guidance during internships

D. Share experience in physical fitness management for enterprise employees

E. Currently unable to provide support

F. Other, please specify: ______

QC14. How would you like vocational colleges to integrate occupational physical fitness training with job demands? Multiple responses are allowed.

A. Customize physical fitness training content according to job types

B. Introduce physical fitness training cases based on real enterprise work scenarios

C. Invite enterprise technical personnel to participate in physical fitness course teaching

D. Other, please specify: ______

QC15. Does your enterprise provide occupational physical fitness training or related activities for information technology employees?

A. Regularly, such as 1–2 times per month

B. Occasionally, such as once per quarter

C. Never

D. Planned

QC16. What efforts has your enterprise made to improve employees’ physical fitness qualities? Multiple responses are allowed.

A. Regularly organizes sports activities, such as fitness activities and team-building sports events

B. Provides physical fitness training resources, such as gyms and online courses

C. Incorporates physical fitness and health content into training courses

D. Encourages employees to exercise independently and provides incentives, such as check-in rewards or subsidies

E. Establishes health examination indicators and physical fitness assessment mechanisms

F. No related measures

G. Other, please specify: ______

QC17. Do you have any other valuable suggestions regarding the cultivation of occupational physical fitness qualities among highly skilled information technology talents in vocational colleges?

Thank you again for your support and cooperation in this survey!
