# Supplementary material for: Perceived occupational physical fitness priorities and a preliminary conceptual framework for information technology students at selected vocational colleges in Shanxi Province, China: An exploratory cross-sectional mixed-methods study
Source: PLoS One. 2026 Nov 10;21(11):e0362309. doi: 10.1371/journal.pone.0362309 (PMC13750592; doi:10.1371/journal.pone.0362309)
Supplement: Supplementary file 2 [file pone.0362309.s004.docx]

**S1 Checklist. Consolidated Criteria for Reporting Qualitative Research (COREQ) Checklist for the Semi-Structured Interview Component**

**for the Semi-Structured Interview Component**

Purpose and scope. This checklist was completed retrospectively to improve reporting transparency for the supplementary semi-structured interview component of the mixed-methods study. It does not imply that procedures not documented in the contemporaneous study-report materials were performed. Where the current manuscript explicitly states that information or source materials are unavailable for retrospective verification, the corresponding COREQ item is reported as “Not documented / unavailable for retrospective verification” rather than being reconstructed.

Status terminology: Reported = directly supported by the current manuscript; Partially reported = some, but not all, information requested by the COREQ item is available; Not documented / unavailable = the surviving study documentation does not support a reliable retrospective answer. COREQ is used here as a reporting checklist, not as a numerical quality score.

| **No.** | **COREQ item** | **What should be reported** | **Status** | **Study-specific response and current manuscript location** |
| --- | --- | --- | --- | --- |
| **Domain 1: Research team and reflexivity** | | | | |
| **Personal characteristics** | | | | |
| 1 | Interviewer/facilitator | Identify which author or researcher conducted the interview(s). | Not documented / unavailable | The identity of the interviewer(s) is not documented in the surviving study-report materials. The manuscript explicitly states that interviewer characteristics could not be reliably reconstructed. Methods 2.3, p. 13, lines 309–316; Limitations, p. 56, lines 1241–1248. |
| 2 | Credentials | Report the interviewer/researcher credentials relevant to the qualitative component. | Not documented / unavailable | Because the interviewer(s) cannot be identified from the surviving documentation, their credentials cannot be linked reliably to the interview component. Methods 2.3, p. 13, lines 309–316; Limitations, p. 56, lines 1241–1248. |
| 3 | Occupation | Report the interviewer/researcher occupation at the time of the study. | Not documented / unavailable | The occupation of the interviewer(s) at the time of data collection is not documented for the qualitative component. Methods 2.3, p. 13, lines 309–316; Limitations, p. 56, lines 1241–1248. |
| 4 | Gender | Report the gender of the interviewer/facilitator. | Not documented / unavailable | Interviewer gender is not documented in the surviving study-report materials and was not retrospectively inferred. Methods 2.3, p. 13, lines 309–316; Limitations, p. 56, lines 1241–1248. |
| 5 | Experience and training | Describe qualitative interviewing/research experience or training of the interviewer(s). | Not documented / unavailable | Qualitative interviewing experience or training of the interviewer(s) is not documented and cannot be reliably reconstructed. Methods 2.3, p. 13, lines 309–316; Limitations, p. 56, lines 1241–1248. |
| **Relationship with participants** | | | | |
| 6 | Relationship established | State whether a relationship existed between interviewer(s) and participants before the study. | Not documented / unavailable | No interview-specific information on pre-existing researcher–participant relationships is available in the surviving documentation. Methods 2.3, p. 13, lines 309–316. |
| 7 | Participant knowledge of the interviewer | Describe what participants knew about the researcher/interviewer and the purpose of the research. | Partially reported | Participants received study information and informed-consent materials and confirmed voluntary participation before interviews, but what they knew about the interviewer personally (e.g., role, goals, reasons for conducting the research) is not documented. Ethics Statement, p. 14, lines 330–334; Methods 2.3, p. 13, lines 309–316. |
| 8 | Interviewer characteristics | Report interviewer characteristics or reflexive information that could influence the interview (e.g., assumptions, interests, potential bias). | Not documented / unavailable | No interviewer reflexivity statement, assumptions, interests, or potential biases were documented in the surviving study-report materials. Methods 2.3, p. 13, lines 309–316; Limitations, p. 56, lines 1241–1248. |
| **Domain 2: Study design** | | | | |
| **Theoretical framework** | | | | |
| 9 | Methodological orientation and theory | State the qualitative methodological orientation or analytic approach underpinning the interview component. | Partially reported | The manuscript states that interview data were analyzed using thematic analysis and cites Braun and Clarke. A broader qualitative paradigm or methodological orientation (e.g., phenomenology, grounded theory, ethnography) is not documented. Methods 2.9, p. 24, lines 570–584. |
| **Participant selection** | | | | |
| 10 | Sampling | Describe how interview participants were selected. | Reported | Interview participants were purposively selected within three stakeholder strata (information technology students, teachers, and enterprise personnel) to capture variation in relevant educational and occupational backgrounds and experiences. Methods 2.3, p. 13, lines 294–301. |
| 11 | Method of approach | Describe how potential interview participants were approached (e.g., face-to-face, telephone, email). | Not documented / unavailable | The interview-specific method of approach is not documented. The manuscript reports overall recruitment channels for the survey, but these should not be assumed to describe the interview approach. Detailed interview-procedure records are unavailable. Methods 2.3, p. 13, lines 294–316. |
| 12 | Sample size | Report the number of participants in the qualitative component. | Reported | Table 1 reports 21 student interviewees, 6 teacher interviewees, and 3 enterprise personnel interviewees (total n = 30). Methods 2.3, p. 13, lines 298–301; Table 1, pp. 25–26. |
| 13 | Non-participation | Report interview refusals or dropouts and reasons, if known. | Not documented / unavailable | Interview-specific refusal, non-participation, and dropout counts are not documented in the surviving study-report materials. Detailed interview-procedure records are unavailable for retrospective verification. Methods 2.3, p. 13, lines 309–316; Limitations, p. 56, lines 1241–1248. |
| **Setting** | | | | |
| 14 | Setting of data collection | Report where interviews were conducted (e.g., workplace, school, online, home). | Not documented / unavailable | The location or mode/setting of the interviews is not documented in the surviving study-report materials. Methods 2.3, p. 13, lines 309–316. |
| 15 | Presence of non-participants | State whether anyone other than the participant and researcher was present during interviews. | Not documented / unavailable | Presence or absence of non-participants during interviews is not documented and cannot be retrospectively reconstructed. Methods 2.3, p. 13, lines 309–316. |
| 16 | Description of sample | Describe important characteristics of the qualitative sample. | Partially reported | Stakeholder group and group-specific interview counts are reported (21 students, 6 teachers, 3 enterprise personnel), and purposive selection across the three stakeholder strata is described. Detailed individual demographic characteristics were not systematically recorded, limiting assessment of within-sample variation. Methods 2.3, p. 13, lines 294–302; Table 1, pp. 25–26; Limitations, p. 56, lines 1235–1240. |
| **Data collection** | | | | |
| 17 | Interview guide | Describe the interview guide/questions or prompts and whether the guide was pilot tested. | Partially reported | The manuscript reports the main interview topic domains: occupational physical fitness status, physical discomfort, training participation, curriculum provision, job demands, and school–enterprise cooperation. The exact interview guide/prompts and any interview-guide pilot testing are not documented. Methods 2.3, p. 13, lines 303–305. |
| 18 | Repeat interviews | State whether repeat interviews were conducted and, if so, how many. | Not documented / unavailable | Repeat interviews are explicitly listed among qualitative procedural details that could not be reliably reconstructed. Methods 2.3, p. 13, lines 314–316. |
| 19 | Audio/visual recording | State whether audio or visual recording was used. | Partially reported | According to contemporaneous study-report documentation, interviews were audio-recorded or documented in written form with participant consent. The manuscript does not document which interviews used each mode, and original audio files are no longer available. Methods 2.3, p. 13, lines 306–316; Limitations, p. 56, lines 1241–1248. |
| 20 | Field notes | State whether field notes were made during or after interviews. | Not documented / unavailable | Original field notes are no longer available, and the surviving documentation does not support a reliable determination of how or when field notes were made. Methods 2.3, p. 13, lines 309–316; Limitations, p. 56, lines 1241–1248. |
| 21 | Duration | Report the approximate duration of interviews. | Not documented / unavailable | Exact interview duration is explicitly identified as unavailable for reliable retrospective reconstruction. Methods 2.3, p. 13, lines 314–316. |
| 22 | Data saturation | State whether and how data saturation/information sufficiency was assessed. | Not documented / unavailable | A formal saturation assessment is explicitly identified as unavailable for reliable retrospective reconstruction; no saturation claim is made. Methods 2.3, p. 13, lines 314–316. |
| 23 | Transcripts returned | State whether transcripts were returned to participants for comment or correction. | Not documented / unavailable | Transcript return is explicitly identified as unavailable for reliable retrospective reconstruction. Methods 2.3, p. 13, lines 314–316. |
| **Domain 3: Analysis and findings** | | | | |
| **Data analysis** | | | | |
| 24 | Number of data coders | Report how many researchers coded the qualitative data. | Reported | Two researchers independently read the interview texts and extracted initial codes; coding discrepancies were resolved through discussion. Methods 2.9, p. 24, lines 573–579. |
| 25 | Description of the coding tree | Provide a coding tree, codebook structure, or sufficiently detailed description of coding hierarchy. | Not documented / unavailable | No formal coding tree or codebook is reported. The manuscript describes initial coding and merging of similar codes into themes, but original coding records are no longer available for retrospective verification. Methods 2.9, pp. 24–25, lines 573–591; Limitations, p. 56, lines 1241–1248. |
| 26 | Derivation of themes | State whether themes were prespecified or derived from the data and describe the derivation process. | Reported | The documented process indicates that researchers extracted initial codes from interview texts, compared and discussed them, merged similar codes, and then developed three main themes. Methods 2.9, p. 24, lines 573–584; pp. 24–25, lines 584–591. |
| 27 | Software | Report qualitative data-management or analysis software, if used. | Not documented / unavailable | No qualitative software is identified in the surviving study-report documentation. The manuscript does not state whether coding/data management was manual or software-assisted. Methods 2.9, pp. 24–25, lines 570–591. |
| 28 | Participant checking | State whether participants provided feedback on transcripts, themes, or findings. | Not documented / unavailable | Member checking/participant checking is explicitly identified as unavailable for reliable retrospective reconstruction. Methods 2.3, p. 13, lines 314–316. |
| **Reporting** | | | | |
| 29 | Quotations presented | Present participant quotations illustrating themes/findings and identify their source appropriately. | Partially reported | Table 1 presents one representative quotation for each stakeholder group (student, teacher, enterprise personnel). Quotations are attributed at group level, not by individual participant identifier. Because original verbatim transcripts are no longer available, quotations cannot be independently rechecked against source transcripts. Table 1, pp. 25–26; Limitations, p. 56, lines 1241–1248. |
| 30 | Data and findings consistent | Demonstrate consistency between presented qualitative evidence and the reported findings. | Partially reported | The manuscript presents three documented themes and corresponding group-level representative quotations and explains how qualitative themes complemented/contextualized/qualified quantitative findings. However, original transcripts and coding records are unavailable, so consistency cannot be independently audited against the full source data. Methods 2.9, p. 25, lines 592–600; Table 1, pp. 25–26; Results/Fig 4, p. 43, lines 907–923; Limitations, p. 56, lines 1241–1248. |
| 31 | Clarity of major themes | Clearly present the major themes in the qualitative findings. | Reported | Three main themes are explicitly listed: (1) occupational physical fitness problems among students and lack of training methods; (2) insufficient institutional resources for occupational physical fitness training; and (3) inadequate alignment between enterprise job demands and school-based training provision. Methods 2.9, p. 24, lines 579–584; Table 1, pp. 25–26. |
| 32 | Clarity of minor themes | Describe diverse cases, divergent perspectives, or minor/subthemes where relevant. | Not documented / unavailable | No separate minor themes, divergent cases, or negative/deviant cases are documented in the surviving study-report materials. The available qualitative record preserves only the three main themes, and the original transcripts/coding records are unavailable for further retrospective analysis. Methods 2.9, pp. 24–25, lines 579–591; Limitations, p. 56, lines 1241–1248. |

**Reference for the COREQ checklist**

Tong A, Sainsbury P, Craig J. Consolidated criteria for reporting qualitative research (COREQ): a 32-item checklist for interviews and focus groups. Int J Qual Health Care. 2007;19(6):349–357. doi:10.1093/intqhc/mzm042.
