# Supplementary material for: Perceived occupational physical fitness priorities and a preliminary conceptual framework for information technology students at selected vocational colleges in Shanxi Province, China: An exploratory cross-sectional mixed-methods study
Source: PLoS One. 2026 Nov 10;21(11):e0362309. doi: 10.1371/journal.pone.0362309 (PMC13750592; doi:10.1371/journal.pone.0362309)
Supplement: Supplementary file 3 [file pone.0362309.s005.docx]

S2 Checklist. STROBE Checklist for the Quantitative Cross-Sectional Component

Completed against: Manuscript

Note. This checklist applies only to the quantitative cross-sectional component; the semi-structured interview component is reported separately using COREQ (S1 Checklist).

| **Section** | **Item** | **STROBE recommendation** | Location in Manuscript | **Reporting status / notes** |
| --- | --- | --- | --- | --- |
| Title and abstract | 1(a) | Indicate the study design with a commonly used term in the title or abstract. | p. 1, Lines 3–6; p. 2, Lines 27–29 | Reported. The title and abstract identify the study as an exploratory cross-sectional mixed-methods study. |
| Title and abstract | 1(b) | Provide an informative and balanced abstract summary of what was done and what was found. | pp. 2–3, Lines 23–60 | Reported. Design, participants, sample size, principal descriptive findings, framework, and major limitations are summarized. |
| Introduction | 2 | Explain the scientific background and rationale for the investigation. | pp. 3–7, Lines 64–156 | Reported. |
| Introduction | 3 | State specific objectives, including any prespecified hypotheses. | p. 7, Lines 157–166 | Reported. Three study objectives are stated. No prespecified confirmatory hypothesis was specified because the study was exploratory. |
| Methods | 4 | Present key elements of the study design early in the paper. | pp. 7–8, Lines 169–194 | Reported. Quantitative-dominant cross-sectional mixed-methods design, strand roles, and integration stage are described. |
| Methods | 5 | Describe the setting, locations, and relevant dates, including recruitment and data-collection periods. | pp. 9–12, Lines 196–291 | Reported. Shanxi Province setting, recruitment channels, survey platform, and data-collection dates (20 Apr–29 May 2025) are stated. No follow-up period applies. |
| Methods | 6(a) | Give eligibility criteria and the sources and methods of participant selection. | pp. 9–12, Lines 196–281 | Reported. Eligibility criteria, convenience/snowball sampling, stakeholder-specific recruitment sources, and invitation procedures are described. |
| Methods | 7 | Clearly define outcomes and other variables of interest; give diagnostic criteria if applicable. | pp. 18–23, Lines 426–542; S2 Table; S3 Table | Reported. Principal questionnaire outcomes, stakeholder-specific background variables, five-point importance ratings, dimension definitions, and reporting rules are described. No clinical diagnostic criteria were used; symptom items are explicitly non-diagnostic. |
| Methods | 8 | For each variable of interest, give data sources and assessment methods; describe comparability when more than one group is included. | pp. 15–24, Lines 354–568; S1 Table; S2 Table; S3 Table | Reported. Questionnaire development, three stakeholder versions, common-item comparability, response scales, score construction, assessment procedures, and content-validity procedures are described. |
| Methods | 9 | Describe efforts to address potential sources of bias. | pp. 9–15, Lines 204–348; pp. 26–30, Lines 604–724; pp. 52–57, Lines 1134–1256 | Reported with residual limitations. Voluntary/anonymous recruitment safeguards and prespecified screening are described; residual selection, clustering, recall, social-desirability, common-method, and nonresponse/data-cleaning biases are acknowledged. |
| Methods | 10 | Explain how the study size was arrived at. | pp. 10–11, Lines 236–267 | Reported. Returned and retained sample sizes are given; the manuscript states that no a priori causal-effect-based sample-size calculation was performed because the study was exploratory. |
| Methods | 11 | Explain how quantitative variables were handled in the analyses, including any groupings and their rationale. | pp. 19–23, Lines 452–542; pp. 26–30, Lines 604–724; S2 Table; S3 Table; S5 Table | Reported. Exercise-duration categories, Likert coding, dimension-score construction, multiple-response denominators, ceiling-effect summaries, and handling of stakeholder-specific/common items are described. |
| Methods | 12(a) | Describe all statistical methods, including methods used to control for confounding. | pp. 26–30, Lines 604–724 | Reported. Descriptive statistics, internal consistency, content validity, Kruskal–Wallis tests, Bonferroni pairwise comparisons, BH-FDR correction, effect sizes, and distributional summaries are described. No confounder-adjusted causal model was prespecified. |
| Methods | 12(b) | Describe methods used to examine subgroups and interactions, if applicable. | pp. 27–30, Lines 627–724 | Reported where applicable. Stakeholder-group comparisons and stakeholder-specific descriptive analyses are described; no formal interaction model was conducted. |
| Methods | 12(c) | Explain how missing data were addressed. | pp. 26–27, Lines 604–650 | Reported. Questionnaires containing unanswered items were excluded; no imputation was performed. Structural multiple-response handling and the QB9 ‘None’ recoding rule are described. |
| Methods | 12(d) | If applicable, describe analytical methods that take the sampling strategy into account. | pp. 9–11, Lines 204–267; pp. 29–30, Lines 690–702 | Partially reported. Convenience/snowball sampling is described. Clustering could not be modeled because college/class/enterprise cluster identifiers were not retained; this limitation is explicitly stated. |
| Methods | 12(e) | Describe any sensitivity analyses. | Not applicable | No sensitivity analysis was prespecified or reported. |
| Results | 13(a) | Report numbers of individuals at each stage of the study. | pp. 10–11, Lines 236–252; pp. 31–32, Lines 743–755; pp. 56–57, Lines 1249–1256 | Partially reported. 2,106 questionnaires were returned, 182 were excluded, and 1,924 were analyzed (1,863 students, 37 teachers, 24 enterprise personnel). Counts for survey access, consent, starts/non-completions, and stakeholder-specific pre-cleaning stages were unavailable. |
| Results | 13(b) | Give reasons for non-participation at each stage. | pp. 10–11, Lines 229–246; pp. 56–57, Lines 1249–1256 | Partially reported. Prespecified exclusion reasons are defined, but historical criterion-specific and stakeholder-specific exclusion counts could not be reconstructed. |
| Results | 13(c) | Consider use of a flow diagram. | Not provided; see pp. 10–11, Lines 236–246 and pp. 56–57, Lines 1249–1256 | A complete participant-flow diagram was not provided because platform-level access/consent/start counts and criterion-specific historical disposition counts were unavailable. |
| Results | 14(a) | Give participant characteristics and information on relevant exposures or potential confounders. | pp. 31–34, Lines 743–757; Table 2 | Reported. Demographic, educational, and professional characteristics are presented by stakeholder group. No prespecified confounder set was used because no causal regression model was fitted. |
| Results | 14(b) | Indicate the number of participants with missing data for each variable of interest. | pp. 26–27, Lines 604–650; Fig. 1 note: p. 34, Lines 772–778; Fig. 2 note: p. 36, Lines 806–811; Fig. 3 note: p. 36, Lines 817–821 | Reported for the final analytical sample. Questionnaires with unanswered items were excluded; no missing responses remained for the reported multiple-response items. |
| Results | 15 | Report outcome-event numbers or summary measures. | pp. 34–42, Lines 761–906; Figs. 1–3; Tables 3–5; S5 Table | Reported. Frequencies/percentages, medians/IQRs, internal-consistency coefficients, content-validity indices, exploratory comparison statistics, and full Likert distributions are presented. |
| Results | 16(a) | Give unadjusted estimates and, if applicable, adjusted estimates and measures of precision. | pp. 34–42, Lines 761–906; Table 5 | Partially reported. Unadjusted descriptive estimates and exploratory rank-based comparison statistics/effect sizes are reported. No confounder-adjusted estimates were planned. Confidence intervals are not reported in the current manuscript. |
| Results | 16(b) | Report category boundaries when continuous variables were categorized. | pp. 19–23, Lines 452–542; Table 2; S2 Table; S3 Table | Reported. Exercise-duration categories, age groups where collected, Likert response boundaries, and other categorical coding rules are specified. |
| Results | 16(c) | If relevant, translate relative-risk estimates into absolute risk for a meaningful time period. | Not applicable | No relative-risk or risk-ratio estimates were calculated. |
| Results | 17 | Report other analyses performed, such as subgroup, interaction, or sensitivity analyses. | pp. 38–42, Lines 858–906; Tables 3–5; S5 Table | Reported where applicable. Internal-consistency/content-validity analyses, exploratory stakeholder comparisons with BH-FDR correction, and full response-distribution analyses are reported. No sensitivity analysis was conducted. |
| Discussion | 18 | Summarize key results with reference to the study objectives. | pp. 44–52, Lines 940–1132; p. 57, Lines 1258–1278 | Reported in the Discussion and Conclusions. |
| Discussion | 19 | Discuss study limitations, including sources of potential bias or imprecision and their likely implications. | pp. 52–57, Lines 1134–1256 | Reported comprehensively, including nonprobability sampling, clustering, small/imbalanced stakeholder samples, self-report bias, measurement limitations, ceiling effects, qualitative auditability, and incomplete historical disposition data. |
| Discussion | 20 | Provide a cautious overall interpretation considering objectives, limitations, multiplicity, similar studies, and other relevant evidence. | pp. 44–57, Lines 940–1278 | Reported. Interpretation is explicitly exploratory; multiplicity, effect size, measurement limits, and prior literature are considered. |
| Discussion | 21 | Discuss the generalisability (external validity) of the study results. | pp. 52–53, Lines 1135–1158; p. 57, Lines 1272–1278 | Reported. The manuscript restricts generalisation beyond the selected Shanxi vocational-education context and small teacher/enterprise samples. |
| Other information | 22 | Give the source of funding and the role of the funders, if applicable. | Not reported in Manuscript | AUTHOR CONFIRMATION REQUIRED. Add a Funding statement (including “no external funding” if accurate) and the role of any funder before final submission. |

*Checklist source: STROBE Statement—Checklist of items that should be included in reports of cross-sectional studies. The checklist is used here only for the quantitative cross-sectional component of the mixed-methods study.*
