# Supplementary material for: Perceived occupational physical fitness priorities and a preliminary conceptual framework for information technology students at selected vocational colleges in Shanxi Province, China: An exploratory cross-sectional mixed-methods study
Source: PLoS One. 2026 Nov 10;21(11):e0362309. doi: 10.1371/journal.pone.0362309 (PMC13750592; doi:10.1371/journal.pone.0362309)
Supplement: Supplementary file 5 [file pone.0362309.s007.docx]

**S1 Table. Cross-Version Mapping of the Common Occupational Physical Fitness Importance-Rating Items**

The three questionnaire versions contained stakeholder-specific sections tailored to students, teachers, and enterprise personnel. However, all cross-stakeholder quantitative comparisons were restricted to a common 12-item importance-rating block. Supplementary S1 Table presents the exact cross-version mapping of this common block and identifies the only systematic wording difference, namely the stakeholder-specific introductory stem.

| **Component / item** | **Teacher version (QA14)** | **Student version (QB15)** | **Enterprise personnel version (QC8)** | **Cross-version comparability** |
| --- | --- | --- | --- | --- |
| **Item block** | QA14 | QB15 | QC8 | Same 12-item core block |
| **Introductory stem** | “Based on the three-level structural model of ‘general physical fitness–occupation-specific physical fitness–psychological fitness,’ please evaluate the importance of the following occupational physical fitness elements for the future career development of information technology students.” | “The table below lists three types of occupational physical fitness qualities required for information technology positions. Based on your learning experience and understanding of future work, please evaluate the importance of each quality.” | “Based on the actual work demands of information technology positions in your enterprise, please evaluate the importance of the following three types of occupational physical fitness qualities and their specific elements for employees’ work performance.” | **Contextually adapted.** Teachers rated importance for students’ future career development; students rated importance based on their learning experience and understanding of future work; enterprise personnel rated importance based on actual job demands and employee work performance. |
| **Response instruction** | “Scoring instructions: Please select one score for each item.” | Same wording | Same wording | Identical |
| **Response scale** | 1 = Not important at all; 2 = Not important; 3 = Moderately important; 4 = Important; 5 = Very important. | 1 = Not important at all; 2 = Not important; 3 = Moderately important; 4 = Important; 5 = Very important. | 1 = Not important at all; 2 = Not important; 3 = Moderately important; 4 = Important; 5 = Very important. | **Identical scale, coding direction, and score meaning** |
| **Dimension A** | General physical fitness | General physical fitness | General physical fitness | Identical |
| **A1 Cardiorespiratory endurance** | Basic physical fitness reserve for maintaining long working hours; affects overall work sustainability and attendance stability. | Basic physical fitness reserve for maintaining long working hours; affects overall work sustainability and attendance stability. | Basic physical fitness reserve for maintaining long working hours; affects overall work sustainability and attendance stability. | **Identical item name and description** |
| **A2 Muscular strength** | Strength of the lower back and core muscles needed to maintain body stability; helps prevent lumbar and cervical disorders and reduce sedentary strain. | Strength of the lower back and core muscles needed to maintain body stability; helps prevent lumbar and cervical disorders and reduce sedentary strain. | Strength of the lower back and core muscles needed to maintain body stability; helps prevent lumbar and cervical disorders and reduce sedentary strain. | **Identical item name and description** |
| **A3 Flexibility** | Range of joint motion and muscle extensibility; facilitates relaxation during work breaks and reduces the risk of muscle strain. | Range of joint motion and muscle extensibility; facilitates relaxation during work breaks and reduces the risk of muscle strain. | Range of joint motion and muscle extensibility; facilitates relaxation during work breaks and reduces the risk of muscle strain. | **Identical item name and description** |
| **A4 Bodily coordination** | Movement fluency and balance control ability; affects equipment operation efficiency and the precision of hand–eye coordination. | Movement fluency and balance control ability; affects equipment operation efficiency and the precision of hand–eye coordination. | Movement fluency and balance control ability; affects equipment operation efficiency and the precision of hand–eye coordination. | **Identical item name and description** |
| **Dimension B** | Occupation-specific physical fitness | Occupation-specific physical fitness | Occupation-specific physical fitness | Identical |
| **B1 Sedentary tolerance** | Muscular endurance and physiological adaptability when maintaining a seated posture for a long period, ≥4 hours; directly affects daily office efficiency and comfort. | Muscular endurance and physiological adaptability when maintaining a seated posture for a long period, ≥4 hours; directly affects daily office efficiency and comfort. | Muscular endurance and physiological adaptability when maintaining a seated posture for a long period, ≥4 hours; directly affects daily office efficiency and comfort. | **Identical item name and description** |
| **B2 Visual load tolerance** | Ability to maintain visual function after prolonged screen viewing, such as programming, design, and debugging; related to work accuracy and eye health. | Ability to maintain visual function after prolonged screen viewing, such as programming, design, and debugging; related to work accuracy and eye health. | Ability to maintain visual function after prolonged screen viewing, such as programming, design, and debugging; related to work accuracy and eye health. | **Identical item name and description** |
| **B3 Fine motor operation endurance** | Endurance of the fingers and wrists during prolonged precise operations, such as keyboard input and equipment debugging; affects code quality and operational accuracy. | Endurance of the fingers and wrists during prolonged precise operations, such as keyboard input and equipment debugging; affects code quality and operational accuracy. | Endurance of the fingers and wrists during prolonged precise operations, such as keyboard input and equipment debugging; affects code quality and operational accuracy. | **Identical item name and description** |
| **B4 Static postural endurance** | Stability of the cervical and lumbar regions while maintaining a working posture; influences occupational health status and related medical costs. | Stability of the cervical and lumbar regions while maintaining a working posture; influences occupational health status and related medical costs. | Stability of the cervical and lumbar regions while maintaining a working posture; influences occupational health status and related medical costs. | **Identical item name and description** |
| **Dimension C** | Psychological fitness | Psychological fitness | Psychological fitness | Identical |
| **C1 Stress resistance** | Psychological resilience in coping with high-intensity work pressure and pressure during critical project periods; affects employee stability and work output. | Psychological resilience in coping with high-intensity work pressure and pressure during critical project periods; affects employee stability and work output. | Psychological resilience in coping with high-intensity work pressure and pressure during critical project periods; affects employee stability and work output. | **Identical item name and description** |
| **C2 Sustained concentration** | Ability to maintain attention for a long period, such as more than two consecutive hours of programming; directly determines the quality of complex task completion. | Ability to maintain attention for a long period, such as more than two consecutive hours of programming; directly determines the quality of complex task completion. | Ability to maintain attention for a long period, such as more than two consecutive hours of programming; directly determines the quality of complex task completion. | **Identical item name and description** |
| **C3 Emotional regulation** | Ability to manage negative emotions such as anxiety, frustration, and irritability at work; affects teamwork and turnover rate. | Ability to manage negative emotions such as anxiety, frustration, and irritability at work; affects teamwork and turnover rate. | Ability to manage negative emotions such as anxiety, frustration, and irritability at work; affects teamwork and turnover rate. | **Identical item name and description** |
| **C4 Emergency response capacity** | Ability to make rapid decisions and execute actions when facing sudden technical problems, such as system failures or cyberattacks; related to handling efficiency and customer satisfaction. | Ability to make rapid decisions and execute actions when facing sudden technical problems, such as system failures or cyberattacks; related to handling efficiency and customer satisfaction. | Ability to make rapid decisions and execute actions when facing sudden technical problems, such as system failures or cyberattacks; related to handling efficiency and customer satisfaction. | **Identical item name and description** |
| **Dimension score: General physical fitness** | Mean of A1–A4 | Mean of A1–A4 | Mean of A1–A4 | Identical item composition and calculation |
| **Dimension score: Occupation-specific physical fitness** | Mean of B1–B4 | Mean of B1–B4 | Mean of B1–B4 | Identical item composition and calculation |
| **Dimension score: Psychological fitness** | Mean of C1–C4 | Mean of C1–C4 | Mean of C1–C4 | Identical item composition and calculation |
| **Higher score indicates** | Greater perceived importance of the corresponding indicator or dimension | Greater perceived importance of the corresponding indicator or dimension | Greater perceived importance of the corresponding indicator or dimension | Same scoring direction |
| **Use in cross-group analyses** | Included | Included | Included | Cross-stakeholder comparisons were restricted to these 12 common items and the three dimension scores derived from them |
| **Stakeholder-specific questionnaire items outside this block** | Teacher-specific questions on teaching experience, curriculum provision, teaching barriers, and related issues | Student-specific questions on learning experience, discomfort, exercise, training participation, and related issues | Enterprise-specific questions on industry context, job demands, employee-related issues, and enterprise involvement | **Not directly comparable and not included in cross-group statistical testing** |
| **Note:** QA14, QB15, and QC8 refer to the common occupational physical fitness importance-rating blocks administered to teachers, students, and enterprise personnel, respectively. The 12 core indicators (A1–C4) had identical indicator names, item descriptions, response categories, coding direction, and dimensional composition across all three questionnaire versions. The introductory stem was intentionally contextualized according to stakeholder perspective: teachers evaluated the items in relation to the future career development of information technology students, students evaluated them based on their learning experience and understanding of future work, and enterprise personnel evaluated them in relation to actual job demands and employee work performance. Dimension scores were calculated as the mean of the four corresponding core indicators within each dimension. Because the introductory stems differed across stakeholder groups and formal measurement invariance was not examined, cross-stakeholder comparisons should be interpreted as exploratory comparisons of context-specific importance ratings rather than as evidence of full cross-group measurement equivalence. Stakeholder-specific items outside the common 12-item block were not subjected to cross-group statistical comparison. | | | | |
