# Supplementary material for: Perceived occupational physical fitness priorities and a preliminary conceptual framework for information technology students at selected vocational colleges in Shanxi Province, China: An exploratory cross-sectional mixed-methods study
Source: PLoS One. 2026 Nov 10;21(11):e0362309. doi: 10.1371/journal.pone.0362309 (PMC13750592; doi:10.1371/journal.pone.0362309)
Supplement: Supplementary file 6 [file pone.0362309.s009.docx]

**Table S3. Operational Definitions, Reference/Recall Periods, Response Categories, and Reporting Rules for Principal Questionnaire Outcomes**

| **Stakeholder** | **Outcome / variable** | **Questionnaire item and exact wording** | **Reference / recall period** | **Response categories** | **Multiple-response item?** | **Denominator in final analytical sample** | **Missing responses, n** | **Scoring / reporting rule** |
| --- | --- | --- | --- | --- | --- | --- | --- | --- |
| Students | Weekly exercise duration | **QB6.** “On average, how much time do you spend on physical exercise each week, such as running, fitness training, or ball games?” | Explicitly refers to average exercise time per week. | Almost no exercise; 1–3 h/week; 3–5 h/week; 5–7 h/week; >7 h/week | No | 1,863 | 0 | Report n (%) for each original category. The questionnaire assessed weekly exercise duration but did not separately measure exercise intensity, frequency, moderate-to-vigorous physical activity, or sedentary time. Therefore, these categories were not used to classify participants as meeting or not meeting established physical-activity recommendations. |
| Students | Frequency of physical discomfort | **QB8.** “After professional practical operations, such as long-term computer use or equipment debugging, do you experience physical discomfort?” | Contextual reference: after professional practical operations. No fixed retrospective recall period was specified. | Frequently; Occasionally; Rarely; Never | No | 1,863 | 0 | Report n (%) for each original category. When “Frequently” and “Occasionally” are combined for descriptive reporting, explicitly identify this as a descriptive combination of questionnaire response categories rather than a validated frequency threshold or period-prevalence estimate. |
| Students | Types of physical discomfort | **QB9.** “If you experience physical discomfort, what are the main symptoms? Multiple responses are allowed. If you do not experience discomfort, please select ‘None.’” | Conditional context of physical discomfort following professional practical operations; no additional fixed retrospective recall period was specified. | Cervical or lumbar soreness; Dry eyes or blurred vision; Soreness or swelling of the wrist or finger joints; Dizziness or difficulty concentrating; Physical fatigue or insufficient endurance; None; Other | **Yes** | 1,863 | 0 | Report n (%) selecting each option, using all 1,863 student respondents as the denominator. Percentages may sum to >100%. The survey platform did not technically enforce mutual exclusivity for “None.” Eighteen respondents selected “None” together with at least one specific symptom. For logical consistency, the specific symptom selection(s) were retained and the contradictory “None” selection was recoded as unselected; no respondent was excluded. Following this consistency recoding, “None” was selected by 553/1,863 respondents (29.68%). |
| Students | Self-reported psychological experiences | **QB10.** “During professional learning or practice, have you experienced any of the following psychological states? Multiple responses are allowed.” | General context of professional learning or practice; no uniform retrospective recall period was specified. | Feeling so anxious before examinations or project deadlines that sleep is affected; inability to concentrate after approximately 2 h of continuous study/programming; irritation or desire to give up when encountering technical problems; overeating or loss of appetite under learning pressure; fatigue or loss of interest in professional learning; nervousness regarding employment/internship performance; emotional difficulty during team disagreements; rarely experiencing any of the above situations; Other | **Yes** | 1,863 | 0 | Report n (%) selecting each option using 1,863 as the denominator. Percentages may sum to >100%. Results are interpreted as endorsement proportions for self-reported experiences and not as clinical diagnoses or period-prevalence estimates of anxiety, insomnia, sleep disturbance, or other psychological disorders. |
| Students | Coping strategies | **QB11.** “When you experience the above psychological stress or negative emotions, what methods do you usually use to relieve them? Multiple responses are allowed.” | Refers to coping when the experiences described in QB10 occur; no separate retrospective recall period was specified. | Exercise/physical activity; talking with friends/family; listening to music/watching videos; continuing to push through without taking action; overeating or other unhealthy methods; deep breathing/meditation; seeking psychological counseling/counselor; Other | **Yes** | 1,863 | 0 | Report n (%) selecting each option using 1,863 as the denominator. Percentages may sum to >100%. Categories represent self-reported coping strategies and should not be interpreted as scores from a validated coping instrument. |
| Students | Overall perceived importance of occupational physical fitness | **QB12.** “Do you think the occupational physical fitness qualities required by information technology students are important for future work?” | Current perception concerning future work; not a retrospective outcome. | Very important; Relatively important; Average; Not very important; Not important at all | No | 1,863 | 0 | Report the original ordinal response distribution as n (%). The item reflects perceived importance and should not be interpreted as a formally measured unmet need. |
| Teachers | Barriers to job-specific physical-fitness course design | **QA7a.** “When designing physical fitness courses for information technology students, what are the greatest difficulties you encounter? Multiple responses are allowed.” | Current/general teaching context; no fixed retrospective recall period. | Lack of specific data on the physical fitness demands of information technology positions; difficulty matching existing physical fitness training methods to professional job scenarios; low student interest in courses integrating professional learning and physical fitness; lack of targeted training equipment; Other | **Yes** | **17 eligible teachers** | **0 among eligible teachers** | QA7a was administered only to teachers whose main teaching field was physical education or physical fitness-related courses. Report n (%) for each option using 17 as the denominator. Percentages may sum to >100%. The remaining 20 teachers were not eligible for QA7a under the questionnaire skip logic and their blank fields are structural/not-applicable responses rather than missing data. |
| Teachers | Frequency of designing job-demand-based training | **QA8a.** “In your courses, have you ever tried to design physical fitness training content based on the job demands of information technology positions, such as lower back training for prolonged sitting or eye relaxation training for prolonged screen viewing?” | Explicit frequency anchor per semester. | Frequently (>3 times per semester); Occasionally (1–2 times per semester); Never; Would like to, but lack references on job-related physical fitness demands | No | **17 eligible teachers** | **0 among eligible teachers** | Report n (%) for each original category using 17 as the denominator. The questionnaire-defined semester-based frequency thresholds should be retained exactly. The remaining 20 teachers were structurally not applicable rather than missing. |
| Teachers | Willingness to conduct enterprise field investigations | **QA9a.** “If given the opportunity, would you be willing to participate in on-site investigations of enterprise information technology positions to understand their actual physical fitness demands and optimize course design?” | Hypothetical/current willingness; no retrospective recall period. | Very willing; Willing; Neutral; Unwilling; Very unwilling | No | **17 eligible teachers** | **0 among eligible teachers** | Report n (%) for each original category using 17 as the denominator. If “Very willing” and “Willing” are combined, state the exact combination; in the present dataset, 15/17 (88.24%) selected one of these two categories. The other 20 teachers were structurally not applicable rather than missing. |
| Teachers | Institutional provision of specialized occupational physical-fitness courses | **QA10.** “Does your institution offer specialized occupational physical fitness training courses for information technology students?” | Current institutional provision; no retrospective recall period. | Yes, course frequency: ___; No; Planned | No | 37 | 0 | Report n (%) for Yes/No/Planned using all 37 teachers as the denominator. If free-text frequency information under “Yes” is analyzed, report it separately rather than inferring a standardized frequency. |
| Teachers | Teaching-resource priorities | **QA20.** “What teaching resources do you think are most needed in occupational physical fitness teaching? Multiple responses are allowed.” | Current perceived resource priorities; no retrospective recall period. | Specialized occupational physical fitness training materials; physical fitness training equipment; professional physical fitness coaches; case database for occupational physical fitness teaching; Other | **Yes** | 37 | 0 | Report exact n (%) selecting each option using 37 as the denominator. Percentages may sum to >100%. These responses represent perceived resource priorities rather than objectively verified resource shortages. For the principal results, training equipment was selected by 29/37 (78.38%), professional coaches by 28/37 (75.68%), and specialized training materials by 22/37 (59.46%). |
| Teachers | Perceived role of enterprises | **QA23.** “What role do you think enterprises should play in cultivating occupational physical fitness qualities among information technology students? Multiple responses are allowed.” | Current opinion/proposed role; no retrospective recall period. | Provide standards for job-related physical fitness demands; participate in occupational physical fitness curriculum design; provide physical fitness training scenarios in enterprise practice; no participation is needed; Other | **Yes** | 37 | 0 | Report exact n (%) selecting each option using 37 as the denominator. Percentages may sum to >100%. Interpret the responses as teachers’ preferred or proposed enterprise roles rather than evidence of actual enterprise participation. If the explicitly negative option “No participation is needed” is selected together with positive enterprise-role options, retain the substantive positive selections and recode the contradictory negative option as unselected for logical consistency; no respondent is excluded on this basis. |
| Enterprise personnel | Observed frequency of physical-fitness-related work problems | **QC6.** “In daily work, how often have you observed that information technology employees, including graduates, are affected at work by physical fitness-related problems?” | Daily work context, but no fixed retrospective period or numerical definition of frequency. | Frequently; Occasionally; Rarely; Never | No | 24 | 0 | Report n (%) for each original category using 24 as the denominator. Do not retrospectively assign numerical thresholds to “Frequently” or “Occasionally.” |
| Enterprise personnel | Perceived consequences associated with insufficient physical fitness | **QC9.** “What are the common problems among information technology employees in your enterprise caused by insufficient physical fitness? Multiple responses are allowed.” | General/current enterprise experience; no fixed retrospective recall period. | Decreased work efficiency; increased frequency of taking leave, such as due to cervical or lumbar problems; increased rate of operational errors; no obvious problems; Other | **Yes** | 24 | 0 | Report exact n (%) selecting each response using 24 as the denominator; percentages may sum to >100%. The results represent enterprise respondents’ perceptions and should not be interpreted as causal evidence that physical fitness produces these outcomes. In the principal results, decreased work efficiency was selected by 13/24 (54.17%), increased frequency of taking leave by 13/24 (54.17%), and increased operational errors by 8/24 (33.33%). Because “No obvious problems” is logically inconsistent with simultaneous selection of specific problems, contradictory negative selections should be recoded as unselected while retaining the specific problem selections; no respondent is excluded. |
| Enterprise personnel | Attention to occupational physical fitness during recruitment | **QC10.** “When recruiting information technology talents, does your enterprise pay attention to their occupational physical fitness-related performance?” | Current/general recruitment practice; no fixed retrospective recall period. | Pays very close attention; Pays some attention; Average; Pays little attention; Pays no attention at all | No | 24 | 0 | Report n (%) for each ordinal category using 24 as the denominator. If “Pays very close attention” and “Pays some attention” are combined, explicitly state the combination; in the current dataset, 5/24 (20.83%) selected “Pays very close attention” and 10/24 (41.67%) selected “Pays some attention,” corresponding to 15/24 (62.50%) combined. Interpret this item only as respondents’ reported recruitment attention and not as evidence supporting fitness-based employment screening. |
| Enterprise personnel | Potential enterprise support for school-based training | **QC13.** “If vocational colleges invite enterprises to participate in occupational physical fitness training for information technology students, what kinds of support do you think enterprises could provide? Multiple responses are allowed.” | Hypothetical willingness/capability; not past participation. | Provide standards for job-related physical fitness demands; participate in the design of physical fitness training courses; provide physical fitness training scenarios in enterprise practice; share experience in physical fitness management for enterprise employees; currently unable to provide support; Other | **Yes** | 24 | 0 | Report exact n (%) selecting each option using 24 as the denominator. Percentages may sum to >100%. Interpret responses as potential forms of enterprise support, not documented previous participation. In the principal results, 10/24 (41.67%) selected providing job-demand standards, 10/24 (41.67%) selected participation in course design, and 12/24 (50.00%) selected provision of enterprise-practice training scenarios. |
| Enterprise personnel | Enterprise provision of occupational physical-fitness training | **QC15.** “Does your enterprise provide occupational physical fitness training or related activities for information technology employees?” | Explicit frequency anchors for the first two response categories. | Regularly (1–2 times per month); Occasionally (approximately once per quarter); Never; Planned | No | 24 | 0 | Report n (%) for each original category using 24 as the denominator. Retain the questionnaire-defined temporal anchors exactly and do not replace them with retrospectively constructed thresholds. |
| Students / Teachers / Enterprise personnel | Importance ratings of the 12 common occupational physical-fitness indicators | **QB15 / QA14 / QC8.** Participants rated A1–A4, B1–B4, and C1–C4. The stakeholder-specific introductory stem differed, whereas the indicator names, descriptions, response options, and coding were identical across the three questionnaire versions. | Current stakeholder evaluation; not a retrospective symptom measure. | 1 = Not important at all; 2 = Not important; 3 = Moderately important; 4 = Important; 5 = Very important | No | Students: 1,863; Teachers: 37; Enterprise personnel: 24 | 0 in each stakeholder group | Report item-level ordinal response distributions and Mdn (IQR) as appropriate. Each dimension score is calculated as the mean of the same four indicators assigned to that dimension. Higher scores indicate greater perceived importance/priority rather than directly measured unmet need. Because the introductory stems were stakeholder-specific and formal measurement invariance was not evaluated, cross-stakeholder comparisons are interpreted as exploratory rather than as evidence of full psychometric equivalence. |
| **Note:** The wording, response categories, and temporal/reference frames shown in this table reproduce the structure of the original questionnaires. Where no fixed retrospective recall period or numerical frequency threshold was specified in the original questionnaire, none was assigned retrospectively during manuscript revision. For multiple-response questions, the denominator is the number of respondents eligible to answer the corresponding item, not the total number of selections; percentages therefore represent the proportion of eligible respondents selecting each response option and may sum to more than 100%. Unless otherwise stated, there were no missing responses among eligible respondents in the final analytical sample because questionnaires containing unanswered items were excluded during data screening.  For the conditional teacher items QA7a–QA9a, only 17 teachers whose main teaching field was physical education or physical fitness-related courses were eligible to respond. The remaining 20 teachers followed the questionnaire skip logic and are classified as structurally not applicable rather than missing.  For response sets containing an explicitly negative option that was intended to be mutually exclusive with substantive positive options, logical consistency checks were applied without excluding participants. For QB9, 18 students selected “None” together with at least one specific physical-discomfort symptom; the specific symptom selection(s) were retained and “None” was recoded as unselected. The resulting “None” count was 553/1,863 (29.68%). The same principle was applied where an explicitly contradictory negative response occurred in other multiple-response items.  The self-developed physical-discomfort and psychological-experience items are descriptive questionnaire measures and should not be interpreted as validated clinical diagnoses or period-prevalence estimates. The weekly exercise-duration item did not assess exercise intensity or frequency and therefore cannot determine adherence to established physical-activity recommendations. For the 12 common importance-rating items, higher ratings indicate greater perceived importance or priority and do not constitute direct evidence of an unmet occupational physical fitness need. | | | | | | | | |
