# Supplementary material for: Perceived occupational physical fitness priorities and a preliminary conceptual framework for information technology students at selected vocational colleges in Shanxi Province, China: An exploratory cross-sectional mixed-methods study
Source: PLoS One. 2026 Nov 10;21(11):e0362309. doi: 10.1371/journal.pone.0362309 (PMC13750592; doi:10.1371/journal.pone.0362309)
Supplement: Supplementary file 7 [file pone.0362309.s010.docx]

**S4 Table. Mixed-Methods Joint Display Linking Quantitative Findings, Qualitative Themes, and Integrated Interpretations**

| **Domain** | **Quantitative finding** | **Qualitative theme/evidence** | **Integrated interpretation** |
| --- | --- | --- | --- |
| Student experiences/training | 68.71% selected “Frequently” or “Occasionally” for physical discomfort; occupation-specific training exposure was limited | Student theme: lack of occupation-specific training methods; representative quotation concerning lack of targeted exercise for eye fatigue | Qualitative findings contextualize the survey pattern by indicating limited knowledge/access to targeted training; they do not establish that a specific intervention would be effective |
| School/teacher provision | Among eligible PE teachers, 13/17 (76.47%) selected lack of job-demand data as a barrier | Teacher theme: insufficient institutional resources; “There is a lack of job demand data...” | Interview evidence provides contextual explanation for the reported curriculum-design and resource constraints |
| Enterprise context | Enterprise respondents identified potential work-related consequences and potential forms of school support | Enterprise theme: inadequate alignment between enterprise job demands and school-based training | Qualitative evidence provides occupational context for enterprise responses but does not establish causal effects or employment-screening validity |
| Stakeholder priorities | Importance ratings were generally high; no comparison remained statistically supported after FDR correction | Interview themes differed in contextual emphasis across stakeholder groups | Quantitative ratings indicate broad perceived importance, while qualitative findings provide contextual differentiation; neither source demonstrates stakeholder equivalence or consensus |
