# Supplementary material for: Perceived occupational physical fitness priorities and a preliminary conceptual framework for information technology students at selected vocational colleges in Shanxi Province, China: An exploratory cross-sectional mixed-methods study
Source: PLoS One. 2026 Nov 10;21(11):e0362309. doi: 10.1371/journal.pone.0362309 (PMC13750592; doi:10.1371/journal.pone.0362309)
Supplement: Supplementary file 8 [file pone.0362309.s011.docx]

**S5 Table. Full Response Distributions for the 12 Occupational Physical Fitness Importance-Rating Indicators by Stakeholder Group**

| **Group** | **Indicator** | **1, n (%)** | **2, n (%)** | **3, n (%)** | **4, n (%)** | **5, n (%)** |
| --- | --- | --- | --- | --- | --- | --- |
| Students | A1 Cardiorespiratory endurance | 76 (4.08) | 23 (1.23) | 494 (26.52) | 636 (34.14) | 634 (34.03) |
| Students | A2 Muscular strength | 66 (3.54) | 33 (1.77) | 443 (23.78) | 643 (34.51) | 678 (36.39) |
| Students | A3 Flexibility | 60 (3.22) | 35 (1.88) | 483 (25.93) | 637 (34.19) | 648 (34.78) |
| Students | A4 Bodily coordination | 60 (3.22) | 33 (1.77) | 439 (23.56) | 626 (33.60) | 705 (37.84) |
| Students | B1 Sedentary tolerance | 59 (3.17) | 20 (1.07) | 497 (26.68) | 606 (32.53) | 681 (36.55) |
| Students | B2 Visual load tolerance | 58 (3.11) | 28 (1.50) | 446 (23.94) | 623 (33.44) | 708 (38.00) |
| Students | B3 Fine motor operation endurance | 58 (3.11) | 36 (1.93) | 466 (25.01) | 623 (33.44) | 680 (36.50) |
| Students | B4 Static postural endurance | 56 (3.01) | 29 (1.56) | 459 (24.64) | 614 (32.96) | 705 (37.84) |
| Students | C1 Stress resistance | 55 (2.95) | 29 (1.56) | 465 (24.96) | 602 (32.31) | 712 (38.22) |
| Students | C2 Sustained concentration | 58 (3.11) | 30 (1.61) | 488 (26.19) | 579 (31.08) | 708 (38.00) |
| Students | C3 Emotional regulation | 55 (2.95) | 28 (1.50) | 461 (24.75) | 596 (31.99) | 723 (38.81) |
| Students | C4 Emergency response capacity | 54 (2.90) | 26 (1.40) | 463 (24.85) | 587 (31.51) | 733 (39.35) |
| Teachers | A1 Cardiorespiratory endurance | 2 (5.41) | 2 (5.41) | 3 (8.11) | 15 (40.54) | 15 (40.54) |
| Teachers | A2 Muscular strength | 1 (2.70) | 1 (2.70) | 5 (13.51) | 14 (37.84) | 16 (43.24) |
| Teachers | A3 Flexibility | 1 (2.70) | 2 (5.41) | 13 (35.14) | 11 (29.73) | 10 (27.03) |
| Teachers | A4 Bodily coordination | 1 (2.70) | 1 (2.70) | 8 (21.62) | 17 (45.95) | 10 (27.03) |
| Teachers | B1 Sedentary tolerance | 2 (5.41) | 0 (0.00) | 0 (0.00) | 19 (51.35) | 16 (43.24) |
| Teachers | B2 Visual load tolerance | 1 (2.70) | 2 (5.41) | 2 (5.41) | 14 (37.84) | 18 (48.65) |
| Teachers | B3 Fine motor operation endurance | 1 (2.70) | 1 (2.70) | 4 (10.81) | 15 (40.54) | 16 (43.24) |
| Teachers | B4 Static postural endurance | 2 (5.41) | 0 (0.00) | 4 (10.81) | 16 (43.24) | 15 (40.54) |
| Teachers | C1 Stress resistance | 1 (2.70) | 1 (2.70) | 4 (10.81) | 12 (32.43) | 19 (51.35) |
| Teachers | C2 Sustained concentration | 1 (2.70) | 1 (2.70) | 1 (2.70) | 14 (37.84) | 20 (54.05) |
| Teachers | C3 Emotional regulation | 2 (5.41) | 0 (0.00) | 3 (8.11) | 16 (43.24) | 16 (43.24) |
| Teachers | C4 Emergency response capacity | 1 (2.70) | 1 (2.70) | 4 (10.81) | 15 (40.54) | 16 (43.24) |
| Enterprise personnel | A1 Cardiorespiratory endurance | 2 (8.33) | 0 (0.00) | 7 (29.17) | 5 (20.83) | 10 (41.67) |
| Enterprise personnel | A2 Muscular strength | 2 (8.33) | 0 (0.00) | 5 (20.83) | 7 (29.17) | 10 (41.67) |
| Enterprise personnel | A3 Flexibility | 2 (8.33) | 0 (0.00) | 6 (25.00) | 8 (33.33) | 8 (33.33) |
| Enterprise personnel | A4 Bodily coordination | 1 (4.17) | 0 (0.00) | 5 (20.83) | 10 (41.67) | 8 (33.33) |
| Enterprise personnel | B1 Sedentary tolerance | 2 (8.33) | 0 (0.00) | 5 (20.83) | 7 (29.17) | 10 (41.67) |
| Enterprise personnel | B2 Visual load tolerance | 2 (8.33) | 0 (0.00) | 5 (20.83) | 7 (29.17) | 10 (41.67) |
| Enterprise personnel | B3 Fine motor operation endurance | 2 (8.33) | 0 (0.00) | 6 (25.00) | 5 (20.83) | 11 (45.83) |
| Enterprise personnel | B4 Static postural endurance | 2 (8.33) | 1 (4.17) | 5 (20.83) | 8 (33.33) | 8 (33.33) |
| Enterprise personnel | C1 Stress resistance | 1 (4.17) | 0 (0.00) | 5 (20.83) | 5 (20.83) | 13 (54.17) |
| Enterprise personnel | C2 Sustained concentration | 1 (4.17) | 0 (0.00) | 5 (20.83) | 5 (20.83) | 13 (54.17) |
| Enterprise personnel | C3 Emotional regulation | 1 (4.17) | 0 (0.00) | 5 (20.83) | 6 (25.00) | 12 (50.00) |
| Enterprise personnel | C4 Emergency response capacity | 1 (4.17) | 0 (0.00) | 6 (25.00) | 4 (16.67) | 13 (54.17) |
| *Note.* Scores ranged from 1 (“Not important at all”) to 5 (“Very important”). Percentages were calculated within each stakeholder group: students, n = 1,863; teachers, n = 37; enterprise personnel, n = 24. The full distributions are presented to permit evaluation of upper-end response concentration and possible ceiling effects. | | | | | | |
